# Treatment scheduling effects on the evolution of drug resistance in heterogeneous cancer cell populations

**DOI:** 10.1038/s41523-021-00270-4

**Published:** 2021-05-26

**Authors:** Gauri A. Patwardhan, Michal Marczyk, Vikram B. Wali, David F. Stern, Lajos Pusztai, Christos Hatzis

**Affiliations:** 1 Breast Medical Oncology, Yale Cancer Center, Yale University School of Medicine, New Haven, CT USA; 2 Department of Data Science and Engineering, Silesian University of Technology, Gliwice, Poland; 3 Department of Pathology, Yale School of Medicine, New Haven, CT USA

**Keywords:** Targeted therapies, Cancer therapeutic resistance, Cancer therapeutic resistance, Breast cancer, Tumour heterogeneity

## Abstract

The effect of scheduling of targeted therapy combinations on drug resistance is underexplored in triple-negative breast cancer (TNBC). TNBC constitutes heterogeneous cancer cell populations the composition of which can change dynamically during treatment resulting in the selection of resistant clones with a fitness advantage. We evaluated crizotinib (ALK/MET inhibitor) and navitoclax (ABT-263; Bcl-2/Bcl-xL inhibitor) combinations in a large design consisting of 696 two-cycle sequential and concomitant treatment regimens with varying treatment dose, duration, and drug holiday length over a 26-day period in MDA-MB-231 TNBC cells and found that patterns of resistance depend on the schedule and sequence in which the drugs are given. Further, we tracked the clonal dynamics and mechanisms of resistance using DNA-integrated barcodes and single-cell RNA sequencing. Our study suggests that longer formats of treatment schedules in vitro screening assays are required to understand the effects of resistance and guide more realistically in vivo and clinical studies.

## Introduction

The prevailing cancer therapeutic paradigm typically involves combinations of drugs with complementary non-overlapping mechanisms of action targeting specific genetic aberrations of the cancer cells^1^. However, after the initial response, resistance often develops resulting in treatment failure and cancer relapse. The drug dose and duration of each treatment cycle, as well as the sequence and time between treatment cycles, can influence how and when drug resistance emerges^2–8^.

Efficient screening for effective targeted combination therapies represents a tremendous challenge due to the combinatorial nature of the problem^9^, which is further complicated by also needing to address the scheduling aspects of the treatments that can impact the emergence and dynamics of resistance. The recent surge of targeted therapies has posed major challenges in developing novel effective combinations and filtering out futile ones. In vitro screens that are designed to evaluate the efficacy of targeted combination therapies while also addressing the potential emergence of resistance would be more likely to translate to early clinical trials^10^.

Triple-negative breast cancer (TNBC) is a clinically aggressive subtype associated with high mortality rates. TNBC is genetically heterogeneous^11^ comprised of subpopulations arising from clonal variants that are genetically linked but potentially having different fitness. The tumor composition can change dynamically during tumor growth and also during treatment, resulting in the selection of treatment-resistant subpopulations with a high potential for metastasis. These subpopulations can be pre-existing (innate) or may evolve during treatment under selection in the therapy-driven microenvironment (acquired)^12^. Thus, treatments can selectively kill dominant tumor subpopulations, but surviving cells can replicate and become dominant, causing tumor relapse. Powerful tools such as single-cell RNA sequencing (scRNAseq) and DNA barcoding can characterize the fitness of individual cell clones relative to other competing clones in the tumor under specific treatment schedules by a phylogenetic deconstruction of resistant clones^13–16^.

Standard in vitro high-throughput drug combination screening (HTS) assays involve cell viability assessment at 2 or 3 days with a single drug or drug combination cocktail bolus treatment in cell lines, which does not probe treatment interactions and long-term effects. Thus, although the standard 3-day assays assess the killing potential of drugs, they may not reflect the effectiveness of treatments in the clinical settings, leading to frequent failure of drugs in clinical trials^17^. Previously, using standard 3-day HTS we identified the combination targeted therapy of crizotinib, an ALK/ROS1 inhibitor, and navitoclax (ABT-263), a Bcl-2/Bcl-xL inhibitor, to be particularly effective and highly synergistic against MDA-MB-231 TNBC cells^9^. To understand the complex interplay between cancer cell growth and selective treatment-induced cell death in a longer format, we performed a 26-day study wherein MDA-MB-231 cells were treated with concurrent or sequential crizotinib and navitoclax. We evaluated the effect of sequential or concurrent administration of the drugs at different doses in two treatment cycles to be more representative of the clinical setting. To more thoroughly assess the dynamics of the process, we varied the duration of each treatment cycle separately (1, 2, or 3 days), and the recovery phase (drug holiday) between the two cycles (2, 5, or 10 days), encompassing a total of 696 treatment conditions. DNA barcoding of these cells enabled us to track the dynamics of clonal selection, and scRNAseq revealed transcriptional patterns linking treatment schedule with the emergence of resistance.

## Results

### Design to systematically evaluate treatment scheduling effects

We tested in excess of 500 treatment schedules utilizing six different regimens of navitoclax/crizotinib to address the following questions: (1) In concomitant administration of the two drugs, how does the dose influence efficacy? (2) Is the sequential administration of the two drugs as effective as concomitant? (3) In sequential regimens, does the sequence in which the two drugs are administered matter? (4) For both the sequential and concomitant administration schedules, how does the duration of the treatment cycles and the duration of the recovery phase influence overall efficacy? (5) How do the different treatment schedules affect the emergence of drug resistance?

**Fig. 1:**
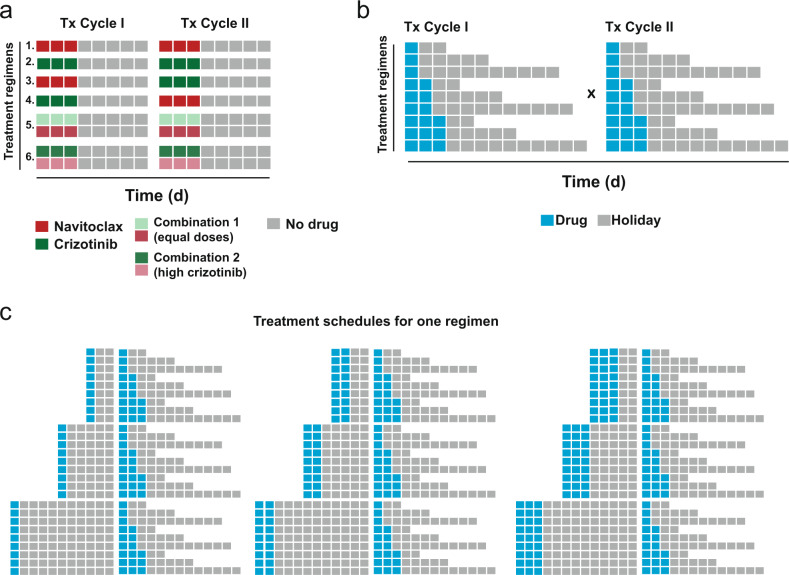
Schematic of in-vitro experimental design to assess the efficacy of drug combinations. **a** Depiction of the six sequential and concurrent treatment regimens tested in this study. A seventh regimen included vehicle treatment. **b** Treatment regimens included two treatment cycles, each comprising of a treatment phase (incubation with the drug) and a drug-free recovery period. **c** Set of 81 treatment schedules evaluated for each treatment regimen.

To systematically address the above questions, MDA-MB-231 cells were treated with seven treatment regimens (Fig. 1a), including single-drug regimens (Regimens 1, 2), sequential administration of the two drugs (Regimens 3, 4), or their concomitant administration (Regimens 5, 6), with the vehicle as non-treatment control. For each regimen, treatment duration of 1, 2, or 3 days and drug-free recovery periods of 2, 5, or 10 days were selected (Fig. 1b), leading to a total of 696 unique treatment schedules with a total duration between 6 days to 26 days for each of the seven regimens (Fig. 1c). IC_90_ doses of the drugs were employed in single drug or sequential regimens (10 μM navitoclax, 8 μM crizotinib), and two different IC_90_-equivalent combinations (1 μM navitoclax/1 μM crizotinib or 0.5 μM navitoclax/2.5 μM crizotinib) were used to eliminate the majority of the drug-sensitive cells (Supplementary Fig. 1a). Viable cell numbers measured at the end of the treatment schedule were greatly affected by the regimen used and by the specific treatment schedule (Supplementary Data 1). For example, in the two-cycle single-agent treatment regimen with navitoclax (10 µM) where cells were treated with the drug for 3 days in each cycle, increasing the recovery periods from 2 days to 10 days caused a 9-fold increase in viable cells at the end of cycle 2. Thus, even varying only the duration of the recovery phase within each treatment cycle, can drastically affect cell survival.

### Drug doses within equivalent concurrent regimens impact overall efficacy

**Fig. 2:**
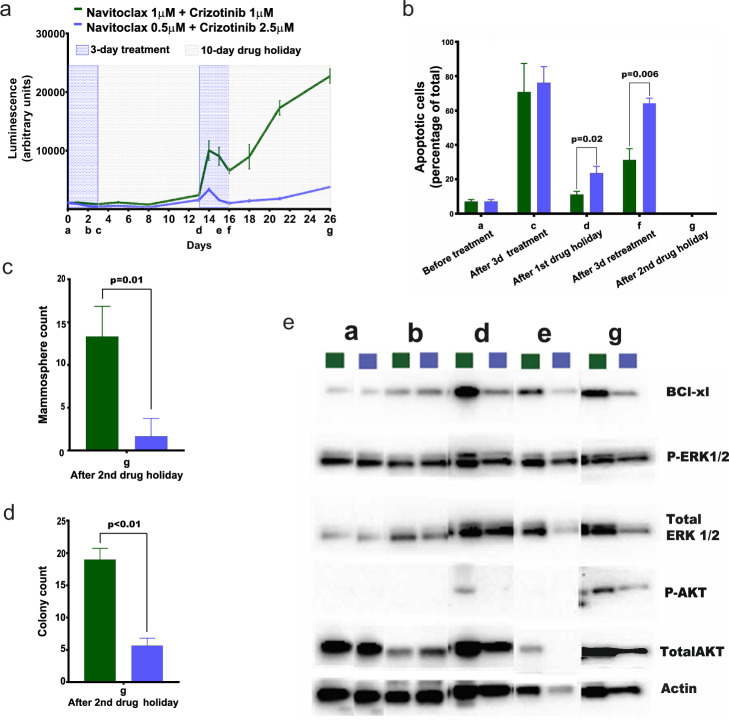
Assessment of concurrent combination treatment regimens. **a** Cell growth curve for two different combination regimens. The schedules included two cycles, each consisting of a 3-day treatment period followed by a 10-day recovery period. Points on time axis: a-pre-treatment baseline, b-after 2 days treatment, c-after 3 days treatment, d-after 10 days of recovery, e-after 2 days of treatment in cycle 2, f-after 3 days of treatment in cycle 2, g-after 10 days of recovery in cycle 2. **b** Percentage of apoptotic cells using Annexin V and PI staining at different treatment points in the schedule. **c** Mammosphere count after the end of the treatment schedule. **d** Colony formation assay after the end of the treatment schedule. Representative pictures of colony formation are provided in Supplementary Fig. 2b. **e** Quantification by Western blot of protein levels involved in targeted pathways. In **a**–**d**, the error bars shown represent the standard deviation of triplicate measurements (biological replicates). *P*-values are from a two-sided *t*-test.

To assess how the dose of each drug in the concomitant administration of the two drugs affects overall efficacy, we selected two different combinations of navitoclax and crizotinib for that both had IC_90_ effect in the standard 3-day assay (combination 1: 1 μM navitoclax/1 μM crizotinib; combination 2: 0.5 μM navitoclax/2.5 μM crizotinib) (Supplementary Fig. 1a). To assess the overall efficacy of the two combinations, we focused on the longest treatment schedule of 26 days that included two cycles of a 3-day treatment phase followed by a 10-day recovery phase (Fig. 2a). This schedule more closely mimics 2-week treatment cycles that are used in many clinical regimens, and the longer cycle time allows regrowth of resistant clones allowing their molecular characterization. Overall, combination 2 with the lower navitoclax dose was the most effective of the two concomitant schedules over two cycles. We notice that in the first treatment cycle, both combinations had a comparable impact on cell viability after 3 days with 32.5 versus 16.8% cells surviving compared to vehicle (Fig. 2a and Supplementary Fig. 2a) and a similar proportion of apoptotic cells, 71 versus 76% (Fig. 2a and Supplementary Fig. 1a), for combination 1 and 2, respectively. Although cells recovered similarly in the first recovery period after the second treatment cycle cells treated with combination 1 continued growing robustly and nearly doubled at the end of the retreatment period compared to those in combination 2 for which growth was controlled (Fig. 2a). After 26 days at the end of treatment, combination 2 was more effective at controlling growth compared to combination 1 that resulted in 6-times greater viable cell mass. Combination 2 treatment also induced a higher rate of apoptosis both after the first drug holiday and after retreatment (Fig. 2b). Flow cytometric analysis using EpCAM, CD24, and CD44 cell surface markers exhibited a similar percentage of stem-like cells with either combination (Supplementary Fig. 2c). We further evaluated mammosphere formation and 2D colony formation as surrogate in vitro markers for tumorigenicity of these stem-like cells at the end of the second recovery period (26th day). At that time, cells that received combination 1 formed significantly more mammospheres (8-fold greater for combination 1; *t*-test *p* = 0.01) and viable colonies (3.35-fold greater for combination 1; *t*-test *p* < 0.01) compared to cells treated with combination 2 (Fig. 2c, d, Supplementary Fig. 2b).

To further probe into the underlying molecular mechanisms explaining the differences in cell response between the two concurrent treatment combinations, we quantified the levels of apoptotic regulators (Fig. 2e). Navitoclax is known to target the antiapoptotic Bcl-2 family members Bcl-2, Bcl-w, and Bcl-xL, although MDA-MB-231 cells express very low basal levels of *BCL2* (Supplementary Fig. 3a) and medium levels of *BCL2L1* (Supplementary Fig. 3b). Upregulation of Bcl-xL has been reported in cells treated with ABT-199, a selective inhibitor of Bcl-2^18^. In our study, Bcl-xL levels were similar until the end of the first treatment for both combinations (Fig. 2e and Supplementary Fig. 1c), but after the first recovery period cells treated with combination 1 displayed elevated levels of Bcl-xL. High Bcl-xL was associated with increased phosphorylated AKT and ERK (normalized to total levels) compared to combination 2 that remained high throughout the second retreatment cycle (Supplementary Data 3). Thus, navitoclax appears to upregulate Bcl-xL via a negative feedback loop, which is associated with higher downstream activity of AKT and ERK mitogenic pathways, rendering cells insensitive to retreatment (Fig. 2e). The higher dose of crizotinib in combination 2 suppresses AKT and ERK phosphorylation resulting in an overall greater tumoristatic effect.

### The order of drugs in sequential regimens could precondition cells for resistance

**Fig. 3:**
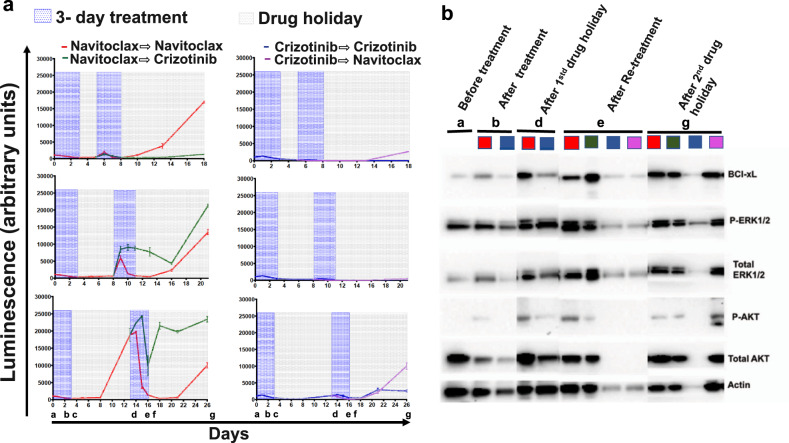
Sequential regimens result in different patterns of resistance. **a** Growth curves associated with sequential treatment regimens and schedules. Cells received a 3-day treatment followed by 2 days (top), 5 days (middle), or 10 days (lower) of recovery in cycle 1, followed by 3-day treatment and 10-day recovery in cycle 2. Left Panel: navitoclax given as cycle 1 treatment. Right panel: crizotinib given as cycle 1 treatment. **b** Quantification by Western blot of protein levels involved in targeted pathways at different times in the treatment cycle. Letters correspond to the time points on the plots in **a**. The colors identifying the different samples at each time point corresponding to the color of the curves in **a**.

Next, to examine the efficacy of sequential navitoclax and crizotinib, we treated cells with one cycle of navitoclax followed by one cycle of crizotinib (Fig. 1a; Regimen 3) and also tested the reverse order of administration of the two drugs (Fig. 1a; Regimen 4) as shown in Fig. 3. Single-agent regimens that included two treatment cycles with the same drug were also included as controls. The left panel in Fig. 3a shows navitoclax as the first cycle treatment in schedules that only vary in the duration of the first recovery period (2, 5, or 10 days). After 2-day recovery cells are sensitive to crizotinib (Fig. 3a, upper left), but with longer recovery periods cells recover exponentially and become resistant to subsequent treatment with crizotinib (Fig. 3a left, middle: 5 days and lower: 10 days). These results are consistent with previous reports that single-agent navitoclax has limited efficacy in human xenografts and patients in a phase II lung cancer study^19,20^. However, these recovered cells were still sensitive to the second cycle of navitoclax retreatment started after 10 days of drug holiday, but interestingly, were resistant to subsequent treatment with crizotinib rendering it an ineffective sequential treatment regimen (Fig. 3a, bottom left). In single-agent therapies, two cycles of crizotinib and in sequential therapies, crizotinib followed by navitoclax were clearly superior treatment regimens that inhibited long-term cell growth (Fig. 3a, right panels). However, at the end of the 10-day drug-free holiday after the second treatment cycle, cells started proliferating again and showed activation of ERK and AKT (Fig. 3b), suggesting the emergence of resistant clones even with this regimen. Bcl-xL overexpression has been implicated in resistance to tyrosine kinase inhibitors^21^, so treatment with navitoclax may have inhibited the apoptotic effect of crizotinib (Fig. 3b). Neither crizotinib, as a single agent, or the crizotinib-navitoclax sequential regimen upregulated Bcl-xL, and these treatments were considerably more effective (Fig. 3b).

These results demonstrate that not only the specific combination of treatments but also the sequence by which they are administered to a large extent determines the durability of the treatment effect and may lead to the emergence of drug resistance. A better understanding of how these phenomena interact with the dynamics of cell growth is crucial for designing effective schedules for administering combination cancer therapies.

### Treatment scheduling influences the evolution of drug resistance

**Fig. 4:**
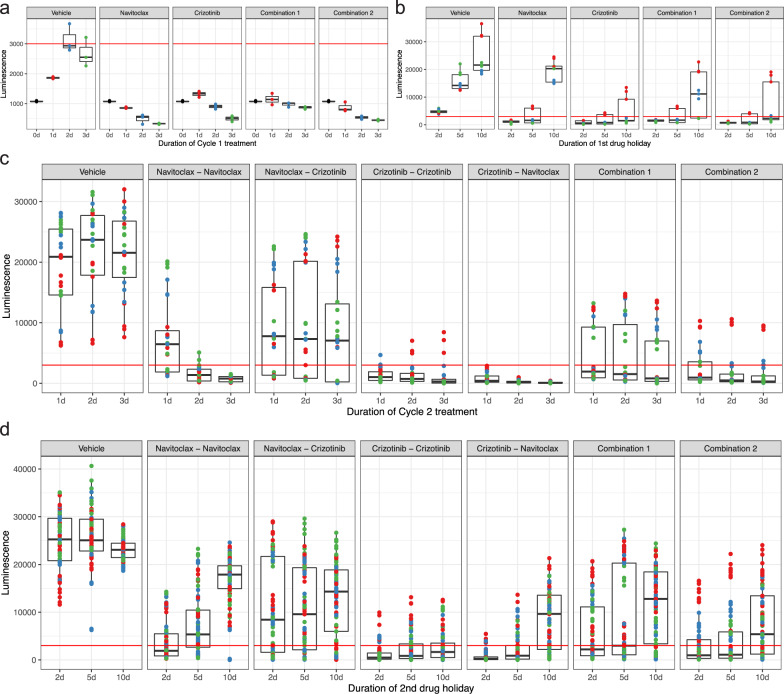
Effect of treatment schedules on cell response. **a** Effect of cycle 1 treatment and duration on MDA-MB-231 cell growth. **b** Effect of the duration of drug-free recovery after cycle 1. Each group corresponds to a different duration of drug-free recovery and includes all cycle 1 schedules for the corresponding treatment regimen. **c** Effect of cycle 2 treatment and duration over all the combinations of cycle 1 treatment durations and drug-free recovery periods. **d** Effect of the duration of recovery after cycle 2, over all the other combinations of prior durations. Combination 1 included equal doses of navitoclax and crizotinib. Combination 2 included a higher dose of crizotinib than navitoclax. Throughout the plots, points have been color-coded based on the duration of cycle 1 treatment (vehicle—black, 1 day—red, 2 days—blue, 3 days—green). Since scales on panels **a** and **b** differ, we added a red reference line at 3000 to make the comparison easier. The box plots represent the distribution in the luminescence value, with the lower and upper sides of the box representing the first (Q1) and third (Q3) quartiles, the thick line representing the median, and the length of the upper and lower whiskers being 1.5(Q3-Q1).

Further, we evaluated the effect of treatment schedule parameters, including drug sequencing and duration of treatments, and the duration of the drug-free recovery periods following each treatment cycle on cell growth. Overall, crizotinib monotherapy and combination 1 had marginal cytostatic effects, but navitoclax monotherapy or combination 2 were cytotoxic at 2 or 3 days (Fig. 4a, Supplementary Data 1, 2). The subsequent drug-free period revealed interesting recovery dynamics. Irrespective of cycle 1 treatment or schedule, cell growth was controlled after 2 days of recovery, or after 5 days of recovery for the longer cycle 1 treatment durations (Fig. 4b). Combination 2 treatment in cycle 1 could adequately control growth even after 10 days of recovery, provided that treatment duration was at least 2 days, but navitoclax monotherapy that appeared equally effective in cycle 1 failed to control cell growth after 10 days of recovery in drug-free media (Fig. 4b), which highlights the risk of obtaining potentially misleading assessments from standard 3-day screening assays.

Next, we considered the combined effect of cycle 2 treatment and duration over all combinations of the preceding phases. The second cycle of navitoclax added after the first navitoclax treatment was effective at controlling the overgrowth observed during the recovery period (Fig. 4c). However, cycle 2 navitoclax treatment was particularly effective when combined with prior crizotinib, which was able to control cell growth even with 2 days of treatment (Fig. 4c). The reverse order (navitoclax followed by crizotinib) was largely ineffective, emphasizing the importance of drug sequence over a broad range of scheduling scenarios.

Finally, considering the duration of the second drug-free period after cycle 2, crizotinib in cycle 1 followed by crizotinib or navitoclax, or combination 2 that included a higher concentration of crizotinib were similarly effective in controlling growth after up to 5 days in drug-free media (Fig. 4d). The two-cycle crizotinib regimen appeared most effective at controlling cell outgrowth after prolonged recovery (Fig. 4c). Overall, undertreatment in cycle 1 appears to be a major factor in the reemergence of resistant cell populations.

Thus, even though 486 treatment schedules out of 696 appeared effective (Supplementary Fig. 4), only 335 treatment schedules were actually effective after considering the effect of the post-cycle 2 recovery phase. Overall, treatment regimens that received navitoclax first or combination 1 were less effective than other treatment regimens, which could be discovered only after considering different treatment and drug holiday durations. It is noteworthy that these differences were not distinguishable with a typical 3-day treatment assay.

### DNA barcoding allows tracking of clones resistant to navitoclax treatment

To investigate whether the cancer cells that survive and proliferate after two cycles of treatment represent pre-existing resistant clones, we used a DNA barcoding strategy to track the clonal lineages longitudinally through the treatment cycles. Treatment can suppress proliferation and promote the death of drug-sensitive cell clones thus debulking the tumor while promoting outgrowth of drug-resistant clones present in a heterogeneous tumor cell population. In addition, rapid feedback responses through the microenvironment can remodel cancer cell phenotypes and support the proliferation of treatment-resistant subpopulations.

We assessed the impact of sequential treatments on the clonal diversity of cancer cells that had been tagged with a high-density DNA barcode library. Navitoclax treated cells demonstrated an interesting dynamic, where cells were highly sensitive to 72 h treatment (Fig. 4a), but fully recovered after 10 days in drug-free media (Fig. 4b). Yet, upon retreatment with the same concentration of navitoclax, post-navitoclax treated cells were as sensitive to the drug as the treatment-naïve cells (Fig. 4c). We initially sought to understand this dynamics behavior. Barcoded MDA-MB-231 cells were treated with navitoclax for 3 days followed by 10-day recovery and subsequently by a second cycle of 3-day navitoclax retreatment and finally by another 10-day recovery period to allow drug-resistant clones to recover and proliferate. The diversity of barcodes was determined before treatment (baseline), after the first treatment cycle, and also after the second treatment cycle. For this, we used two vials of barcoded cells to capture the variability between vials and two replicates for each of three-time points per vial to capture the variation between the emergence of resistant clones. Thus, for each time point, we collected a total of four samples (Supplementary Fig. 5a). If resistant clones pre-existed and were subsequently selected, we would expect a subset of barcodes present in the baseline sample to be enriched in the post-treatment populations consistently in all replicates. Otherwise, the surviving populations may consist of clones that randomly escaped the cytotoxic effects of the treatment, and in this case, we would observe different clones enriched in each of the four replicates of the post-treatment samples. Interestingly, the highest concordance of unique barcodes recovered, and their abundance was observed between replicates, and then between vials (Supplementary Fig. 5b). Overall, we observed higher concordance between samples from vial 1 than between samples from vial 2. For all subsequent analyses, we merged the four replicates for each of the three treatment samples but excluded barcodes that were present only in a single replicate (Supplementary Fig. 5c). From this point, we coded the groups of barcodes according to their existence in 3 subsequent time points.

**Fig. 5:**
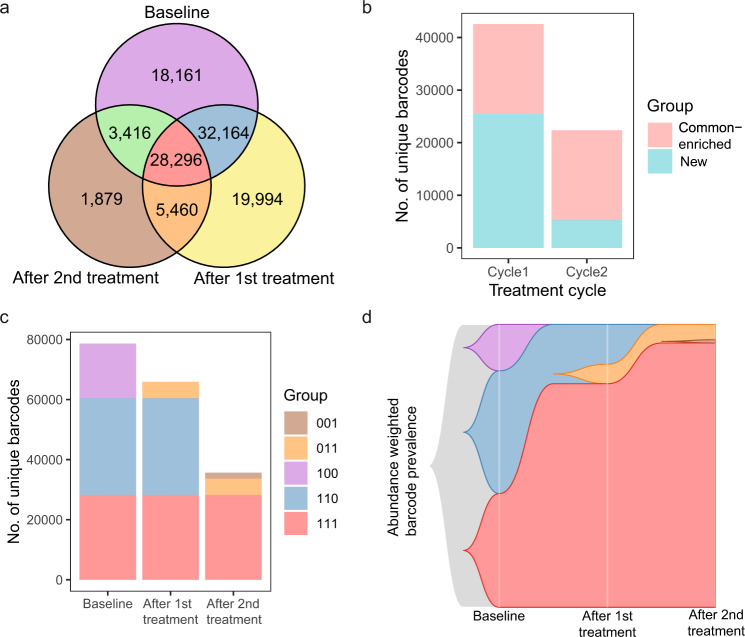
Navitoclax treatment selects pre-existing resistant clones. **a** The number of common unique barcodes between baseline, post-treatment cycle 1, and post-treatment cycle 2 samples. **b** The number of new and existing clones that increased in abundance as detected at end of each cycle: Cycle 1— Post 1st treatment versus Baseline; Cycle 2— Post 2nd treatment versus Post 1st treatment. **c** The distribution of barcodes divided into the 5 subgroups defined in panel **a**, shows a considerable decrease in barcode complexity after treatment. Groups are labeled by a sequence of three numbers representing inclusion (1) or exclusion (0) in the baseline, post-cycle 1, and post-cycle 2 samples. **d** Clonal evolution of cancer cells estimated by the observed barcode prevalence weighted by the abundance of each barcode.

Overall, 28,296 (26%) out of 109,370 total unique barcodes detected were present in all three samples (Fig. 5a). These represent the resistant cell clones that pre-existed in the baseline population and survived both cycles of navitoclax treatment. In addition, 5460 unique barcodes (5%) were observed post-cycle 1 and post-cycle 2 but not at baseline, and 1879 barcodes (2%) observed only post-cycle 2 but not in the prior samples. These clones represent low abundance pre-existing clones that were likely underrepresented in the high clonal complexity baseline sample and thus were not detected. Subpopulations consisting of 18,161 (17%) and 32,164 (29%) unique clones represent navitoclax sensitive cells killed after cycle 1 and cycle 2, respectively (Fig. 5b). Interestingly, although about half of the susceptible clones were killed after one cycle of navitoclax, the remaining required a second cycle to be eliminated. Another set of 19,994 barcodes was observed only after cycle 1 treatment. Cells from these clones started to regrow in the recovery period after cycle 1 treatment (Fig. 3a) and were subsequently killed in cycle 2. Because many of these barcodes were detected only in experiment 2 (samples S5a and S5b, Supplementary Fig. 6c), it is possible that they may be PCR amplification artifacts and were eliminated from further analysis.

Baseline populations shared more clones with those post treatment cycle 1 (Jaccard index = 0.56) compared to populations after cycle 2 (Jaccard index = 0.35), as additional clones were eliminated in cycle 2 (Fig. 5a, b). While 22% of the clones present at baseline were eliminated post-cycle 1, about a third of the remaining clones represent aggressive resistant clones that more than doubled in abundance between baseline and post-cycle 1 (Fig. 5b). However, the majority of the clones that increased in abundance between baseline and post-cycle 1 were new clones (Fig. 5b). In contrast, considerably fewer clones increased in abundance post-cycle 2, and the majority were also present post-cycle 1 (Fig. 5b). Overall, we observed a decrease in barcode complexity after each treatment cycle, with the complexity more drastically reduced after the second cycle to about 50% of the original barcodes (Fig. 5c). The majority of the clones that survive both treatment cycles are resistant clones observed also in the baseline population (Fig. 5c). When the relative abundance of the clones is accounted for, these pre-existing resistant clones that represented about 40% of the initial population became the vast majority (93%) of the resistant population after cycle 2 (Fig. 5d). It appears that a large subset of resistant cells pre-existed, but it is possible that more than one unique barcode could have infected cells originating from the same resistant clone, and therefore we cannot assume that unique barcodes necessarily represent unique clones.

### Single-cell RNA sequencing identifies pathways of resistance to navitoclax treatment

To better understand the mechanisms of resistance, we characterized the transcriptional pathways of cells subjected to the same 2-cycle sequential navitoclax treatment using single-cell RNAseq. We measured expression in approx. twenty thousand cells collected at four time points: before treatment (baseline; S1), on day 3 of cycle 1 treatment (post-treat cycle 1; S2), just prior to the beginning of cycle 2 (pre-cycle 2; S3), and on day 3 of cycle 2 (post-treat cycle 2; S4).

**Fig. 6:**
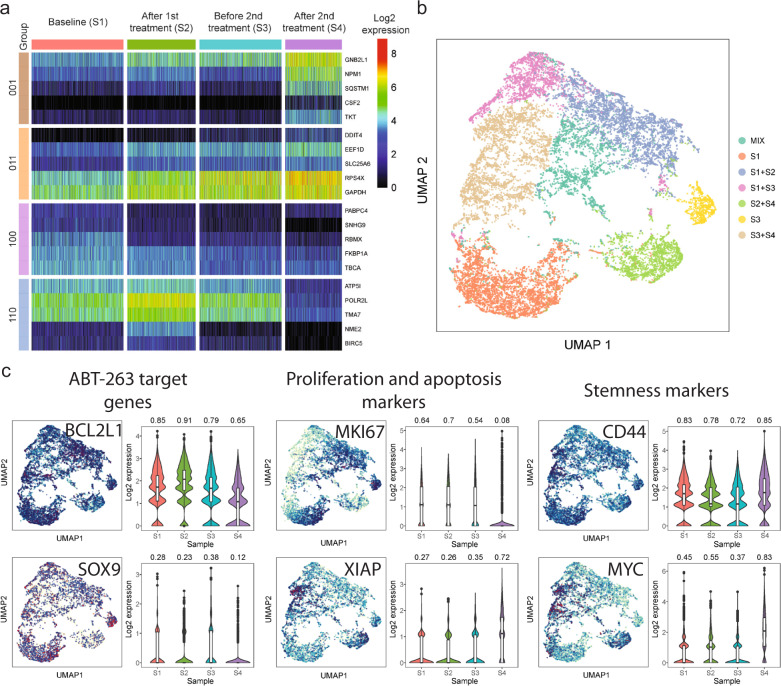
Effects of navitoclax treatment at the single-cell level. **a** Expression heatmap of top five markers of 4 important cell groups defined in DNA-barcoding experiment. **b** UMAP plot presenting cell clustering after alignment of the samples. The name of each cluster represents the dominance of cells from particular samples. **c** Expression of selected important genes presented on UMAP plot (left side) and summarized on boxplot (right side). Above each boxplot, there is a proportion of expressed cells per sample.

To identify transcriptional markers associated with the resistant subgroups detected by DNA barcoding, we compared gene expression in cell groups as follows. To identify markers for cells that survived the last treatment cycle (labeled as 001 in Fig. 6a, where 0 denotes absence and 1 presence and the three numbers represent baseline, post-cycle 1, and post-cycle 2 samples, respectively), we compared the expression levels of cells in the post-cycle 2 sample (S4) vs. those in previous time points (S1, S2, S3). Expression profiles of the top five markers with the highest effect size are presented in Fig. 6a. Among the identified markers overexpressed in the resistant cells included genes *GNB2L1*, *NPM1*, *CSF2*, *TKT* that act as a sensor of the stress response (Fig. 6a). Similarly, to find markers for cells that were present in post-treatment samples but not in baseline (group labeled as 011), we compared all post-treatment samples (S2, S3, S4) versus the baseline sample (S1). Genes involved in protein synthesis and metabolism were identified (*EEF1D*, *RPS4X*, *GAPDH*) that underline a greater proliferative state. Also, we identified genes that were previously reported as markers of resistance of other treatments, e.g., *DDIT4* was responsible for resistance to neoadjuvant chemotherapy in TNBC^22^, and *RPS4X* was found as a marker of cisplatin resistance in two breast cancer cell lines^23^. Similarly, by comparing cells present at baseline but not in any post-treatment samples (group labeled as 100) we identified markers associated with navitoclax sensitive cells, including *RBMX, RKBP1A,* and *TBCA*. Interestingly, among the genes underexpressed in the cells that required the second treatment cycle in order to be eliminated (labeled as 110) was *BIRC5* that encodes for the protein also called survivin, a member of the inhibitor of apoptosis (IAP) gene family that inhibit caspase activation and negatively regulate apoptosis.

Clustering of the 20,000 cells integrated from 4-time points revealed seven distinct groups of cells (Fig. 6b). The middle of the UMAP plot shows the mix of resistant cells observed at all time points. Three clusters were separated from the rest: (i) the orange cluster (bottom left) consisted mostly of highly sensitive cells that were killed after a single cycle of treatment; (ii) the green cluster (bottom right) consisted of cells under stress observed only immediately at the end of treatment cycle 1 and treatment cycle 2; (iii) the yellow cluster (right) consisted mostly of highly proliferating clones that emerged after the recovery phase in cycle 1 and were subsequently killed by the second cycle of treatment.

Figure 6c shows the expression profiles of selected groups of genes, specifically targets of navitoclax, proliferation markers, and apoptosis and stem-like genes. Interestingly, we found that navitoclax reduced expression of *BCL2L1*, but only after cycle 2. We observed the same effect on the indirect target *SOX9*. Also, in most of the cells, after cycle 2 proliferation is significantly reduced, as reflected by the cell proliferation marker *MKI67*, while at the same time *XIAP* an inhibitor of apoptosis is increased further reducing apoptosis and promoting proliferation. After retreatment, we observed an increase in expression of two common stem-cell markers *CD44* and *MYC*, which indicates that the surviving cells show increased resistance to therapy and potentially greater metastatic potential.

Lastly, we found genes that were differentially expressed in subsequent pairs of treatment phases, namely baseline versus post-treatment cycle 1, post-treatment cycle 1 versus post-recovery cycle 1, and post-recovery cycle 1 versus post-treatment cycle 2. The top five differentially expressed genes for each comparison (|logFC| >1 and highest *p*-value) are shown in Supplementary Figs. 7a, b. Among these genes, we noticed some of the markers of cell sensitivity or resistance discussed above. Changes in expression of all genes on a pathway level are summarized in Supplementary Fig. 7c. Late effects of cancer treatment, mostly prevalent after the second cycle of treatment, included a marked increase in inflammatory response pathways, TNFa signaling pathway, and decrease in oxidative phosphorylation. We also observed a decrease in expression of genes involved in the G2M checkpoint and E2F target pathways, which were consistent with reduced proliferation after cycle 2 treatment.

## Discussion

Recent clinical advances have underscored the potential impact of different treatment regimens involving combinations of targeted cancer drugs on improving the survival outcomes of cancer patients^24–28^. Rigorous in vitro evaluation of various drug combination regimens can help identify effective regimens before entering first-in-human studies, thereby increasing the success of clinical trials. Here, we characterize the efficacy of treatment regimens involving the targeted drugs navitoclax (ABT-263) and crizotinib, and systematically address the effect of scheduling variables such as concurrent or sequential administration, order of sequencing, treatment time, and recovery period, can have on the efficacy of the regimen and emergence of resistance in the long-term format in vitro assays. Treatment of DNA barcoded cancer cell populations revealed enrichment of specific clones, indicating that navitoclax treatment selects pre-existing resistant clones. Furthermore, single-cell RNAseq analysis identified genes and pathways associated with resistance mechanisms, which are masked by standard bulk RNAseq analysis.

In vitro treatment studies in long format can provide valuable insight into the interplay between cell kill and growth of resistant clones to help design optimal dosing regimens and combinations that can better predict long-term response and potential resistance in vivo or in clinical studies. Here, we present an extensive assessment of treatment schedule variations (696 schedules) of two targeted therapies, navitoclax and crizotinib, and demonstrate their impact on the final outcome of the treatment. The 26-day treatment schedule encompassing two cycles of treatment followed by drug-free recovery revealed that 335 of the 696 treatment schedules tested were effective, with the majority of them involving crizotinib alone. Interestingly, concurrent combination treatment (Combination 2) with lower doses of crizotinib and navitoclax (~IC_50_) achieved a similar effect as a high dose (IC_90_) crizotinib monotherapy, suggesting that concurrent administration of Combination 2 may deliver better results in mice or in the clinic than either monotherapy or sequential administration of these two drugs.

Our study shows that although navitoclax alone imparts a significant cytotoxic effect, surviving cancer cells recover quickly after long drug holidays, which is reflective of the rapid adaptation of surviving clones to fast-growing clones. Similar to our study, a recent study also reported a reversible drug-tolerant state in TNBC patients and PDX mouse models using DNA barcoding and genomic analysis^29^. Such an effect was not observed with low dose of navitoclax in combination with crizotinib or with crizotinib alone. This can be explained by the different mechanisms of action of the two targeted treatments. We showed that navitoclax treatment causes upregulation of Bcl-xL via negative feedback regulation resulting in higher downstream activity of the AKT and ERK mitogenic pathway, rendering cells insensitive to retreatment. On the other hand, in Combination 2, a higher dose of crizotinib suppresses AKT and ERK phosphorylation leading to cell death without priming cells for resistance.

Tumor heterogeneity is a complex phenomenon that contributes to the development of resistance to treatment. Recent studies have focused on characterizing the evolutionary dynamics of heterogeneous tumors resulting after the treatment of cancers with combination therapies^30–34^. Several studies have shown that heterogeneous tumor cell populations contain resistant tumor clones prior to therapy that are gradually selected as susceptible clones are killed^35–37^. Tumor growth can be controlled by moderating natural competition between susceptible and resistant cells by managing drug dosage or timing more closely to prevent selection or biological adaptation to treatment. For example, Shaw et al reported that a crizotinib-resistant tumor became susceptible to crizotinib retreatment in an NSCLC patient underscoring the importance of treatment scheduling and monitoring of tumor sensitivity^38^. Enriquez-Navas et al. demonstrated that a treatment schedule consisting of a standard high dose of paclitaxel until tumors started to shrink followed by a reduced dose improved treatment-free survival in mice with MDA-MB-231/luc and MCF-7 xenografts. In contrast, in mice that received the standard treatment of a fixed maximum tolerated dose of paclitaxel, tumors grew immediately after stopping treatment^39^. This approach of adaptive therapy was also successful in a pilot study in metastatic castrate-resistant prostate cancer patients with an increase in the median time of disease progression from 16.5 months to 27 months and a reduction in total drug administered by 47%^40^.

We employed DNA barcoding and scRNAseq to track individual cell clones initially present in a cancer cell population and how they evolve under the selection pressure imposed by two cycles of treatment^41^. DNA barcode infected cells exhibited enrichment of the same set of barcodes after treatment and retreatment with navitoclax, indicating selection of pre-existing resistant clones. However, due to the high complexity of the DNA barcode library, we could not capture the presence of exactly the same pre-existing resistant clones between replicates. A similar issue was observed in replicate mice in a recent study by Echiverria et al. using patient derived xenografts of treatment-naive metastatic TNBCs where clonal selection and specific clone dominance in metastases in diverse organs was established using high complexity DNA barcode library^42^. Furthermore, scRNAseq helped to track differential gene expression in baseline cells versus those persisting post-treatment and post-retreatment to understand transcriptional changes associated with drug resistance. Treatment naïve cells at baseline expressed navitoclax sensitivity genes whereas cells that survived after navitoclax treatment expressed genes associated with stress response. Also, all post-treatment samples expressed genes involved in protein synthesis and metabolism indicating greater proliferation.

A limitation of our study is that the combination of crizotinib and navitoclax was assessed in a single TNBC cell line. However, extensive screening performed in our prior study^9^ demonstrated that this combination is synergistic in most TNBC cell lines tested. The main focus of this study was to show that strong efficacy in preclinical testing may not guarantee generalizability and translatability in the clinical setting. Our study illustrates that treatment scheduling is potentially a critical consideration for maximizing drug benefit to the patient and controlling the development of drug resistance. Drug resistance is a dynamic population phenomenon, which is not captured by the standard screening assays with bolus administration of a drug cocktail and 3-day efficacy endpoint assessment. Dynamic treatment scheduling in a set of cancer cell lines would be necessary to help identify the best treatment combination and the best administration schedule to control long-term tumor growth and prevent the establishment and outgrowth of resistant clones. Quantitative modeling of in vitro treatment scheduling data to predict drug response could help direct the selection of rational therapeutic regimens from available targeted therapies for individual patients and enable understanding of disease progression^43^. Furthermore, in vivo studies in PDX mouse models, combining DNA barcode and scRNAseq can help guide combination treatment strategies in patients who develop resistance. Nevertheless, the selection of pre-existing resistant clones is one of the potential mechanisms of drug resistance. Our study showed that treatment with one drug can induce biological changes in the cells that make them more resistant to the other drug. Resistance could even be mediated by cell-to-cell interactions with certain clones influencing the growth of other clones by secreting growth or inhibitory factors in the presence of the drug^14,44,45^. Next-generation drug screening assays and cancer model systems would need to capture some of these nuances in order to be able to address the all so common occurrence of drug resistance^46^.

## Methods

### Cell culture and drugs

The TNBC cell line MDA-MB-231 was purchased from the American Type Culture Collection (Manassas, VA) and maintained in RPMI 1640 media supplemented with 10% FBS and 1% penicillin-streptomycin. The cell line was authenticated by ATCC by short-tandem repeat profiling, karyotyping, morphology, and cytochrome C oxidase I testing. The cell line was used at passages 3 to 9, and cultured less than 3 months after thawing. Crizotinib (catalog no. S1068) and navitoclax (ABT-263 catalog no. S1001) were ordered from Selleckchem, TX.

### Treatments and cell viability in 96-well plates

The IC_90_ dose for monotherapies or concomitant regimens was calculated by treating MDA-MB-231 cells with either navitoclax, crizotinib, or their combination for 72 h in triplicate assays. The dose at which 90 percent of cells were killed was selected as the IC_90_ dose. At least three independent experiments were performed to calculate the IC_90_ dose (Supplementary Fig. 1a). MDA-MB-231 cells were subjected to 696 different treatment schedules resulting by varying treatment duration (1, 2, or 3 days) and drug-free recovery (2, 5, or 10 days) for the first and second treatment cycles independently over a 26-day period (Fig. 1). We used 6 treatment regimens where crizotinib and navitoclax were administered either sequentially (single agents at IC_90_ dose) or concomitantly (combination at IC_90_ dose): (i) crizotinib (8 µM) followed by crizotinib (8 µM); (ii) crizotinib (8 µM) followed by navitoclax (10 µM); (iii) navitoclax (10 µM) followed by navitoclax (10 µM); (iv) navitoclax (10 µM) followed by crizotinib (8 µM); (v) crizotinib (1 µM) + navitoclax (1 µM) followed by crizotinib (1 µM) + navitoclax (1 µM); (vi) crizotinib (2.5 µM) + navitoclax (0.5 µM) followed by crizotinib (2.5 µM) + navitoclax (0.5 µM) and (vii) vehicle control. All treatment schedules were assessed in triplicate at the same time in parallel by using forty-five 96-well plates.

To help interpret the effect of a varying number of cells entering cycle 2 treatment depending on the regiment and schedule in cycle 1, we had control plates to establish standard curves with initial cell numbers ranging from 0–100,000 cells per well for all 6 treatment regimens. For the standard assays, 2000 MDA-MB-231 cells per well were plated in clear bottom opaque-walled 96-well plates (Thermo Scientific, MA) (day 0) and allowed to attach overnight. The following day cells were treated with either crizotinib, navitoclax, or one of the combined regimens as specified for cycle 1 and incubated in the drugs for 1–3 days. At the specified treatment endpoint, the drug-containing media was removed and replaced with drug-free fresh media where cells were incubated for 2–10 days. At each drug holiday endpoint, media were removed again and fresh media with drugs or drug combinations as specified for cycle 2 treatments were added followed by incubation for 1–3 days and followed by a drug holiday of 2–10 days. At the final endpoint of each treatment schedule, viable cell count was determined by incubating cells with CellTiter-Glo reagent (Promega, Madison, WI) for 10 min followed by measuring luminescent ATP using a Synergy HT microplate reader.

### Flow-cytometry

For flow cytometry analysis 350,000 cells were plated in 10 cm plates in triplicate. Cell number was extrapolated from 96 well to 10 cm plate by the surface area of the plate. Apoptotic cells were evaluated using the FITC Annexin V kit (BD Pharmingen, catalog no. 556570) following the manufacturer’s protocol. Briefly, adherent and floating cells after 48 h of treatment were centrifuged for 5 min at 1200 rpm, washed twice with PBS, and resuspended in 1× binding buffer at a concentration of 1 × 10^6^ cells/ml. Then, 100 µl of cell suspension was incubated with 5 µl of FITC Annexin V and 5 µl of PI in binding buffer for 15 min at room temperature in the dark and finally 400 µl of 1× binding buffer was added. Samples were analyzed by 10,000 events per sample excluding doublets and apoptotic cells were determined by measuring FITC-Annexin V and PI fluorescence using an LSRII flow cytometer (Becton-Dickinson) and FlowJo software (version 7.6.5).

Gating strategy—Starting live population was identified by gating FSC/SSC and doublets were excluded by SSC-H/SSC-W and FSC-H/FSC-W. Cells were then identified as apoptotic cells that are Annexin V positive/PI negative and Annexin V-PI double positive. Gates were applied using unstained cells as controls (Supplementary Fig. 8a).

Cancer stem-like cells were evaluated by staining cells for breast cancer cell surface markers EpCAM-FITC, CD24-PE, and CD44-APC at given time points. EpCAM^+^CD44^+^CD24^−^ cells were identified as stem-like cells. Briefly, cells were trypsinized and washed with PBS. Cells were then spun down and approximately 1 × 10^6^ cells were resuspended in 100 μl of blocking buffer (1× DPBS + 2% BSA) for 5 min. Subsequently, 20 µl of each of primary antibodies, APC Mouse Anti-Human CD44 (Cat. # 560890 BD Pharmingen), PE Mouse Anti-Human CD24 (Cat. # 560991 BD Pharmingen) and ESA/Ep-CAM, Mouse MAb anti-Human (Cat. # BMDMM101416 accurate chemical and scientific corporation) were added to cells and gently mixed. Cells were incubated with the fluorescent-labeled antibodies for 20 min at room temperature mixing at every 5 min interval and then quenched with 1 ml Blocking Buffer and spun down at 1200 rpm for 5 min. The supernatant was aspirated and cells were resuspended in 1000 µl of PBS keep on ice until analyzed by flow cytometry and subsequently analyzed by LSRII flow cytometer.

Gating strategy—Starting live population was identified by gating FSC/SSC and doublets were excluded by SSC-H/SSC-W and FSC-H/FSC-W. Cells were then identified as EpCAM-FITC negative (below 10^3^) EpCAM-FITC negative (beyond 10^3^). Basal cells were identified as EpCAM− CD44 + CD24− from EpCAM negative cell population. Stem-like cells were identified as EpCAM + CD44 + CD24− and luminal cells were identified as EpCAM + CD44 + CD24+ from EpCAM positive cell population. Gates were applied using IgG and unstained cells as controls (Supplementary Fig. 8b).

### Mammosphere assay

At the end of the 26-day treatment schedule, cells were trypsinized and 100 cells per well were added in 6 well ultra-low attachment plates in triplicate. By observing under the microscope, spheres that had more than 50 cells at the end of 3 weeks were counted as mammospheres.

### Colony formation assay

At the end of the 26-day treatment schedule, cells were trypsinized and 100 cells per well were added in a 6-well plate in triplicate. Media was changed every 3 days and colonies were observed under the microscope. At the end of 15 days, media was removed, colonies were washed with PBS and then fixed using a 3:1 mixture of methanol/acetic acid for 5 min. Crystal violet solution in methanol (5%) was added to plates for staining. After 15 min, excess staining was removed by washing stained colonies with PBS 5 min for 5 times.

### Immunoblotting

Cells from control and treatment plates for all samples were collected from the same experiment and processed in parallel. Briefly, whole protein lysates were extracted with RIPA buffer containing proteinase and phosphatase inhibitors. Lysate samples were electrophoresed in 4–12% NuPAGE SDS-polyacrylamide midigels (Life Technologies Corporation) and transblotted onto PVDF membrane simultaneously. PVDF membranes were blocked with 2% BSA in 10 mM Tris-HCl,50 mM NaCl, 0.1% Tween 20, pH 7.4 (TBST), followed by incubation with primary antibodies, diluted 1:1000 to 1:5000 in TBST/2% BSA overnight. Bcl-xL (dilution 1:1000, Cat. # 2764), phosphor p44/42 MAPK (dilution 1:2000, Cat. # 4370), p44/42 MAPK (dilution 1:1000, Cat. # 4695), phosphoAKT (dilution 1:2000, Cat. # 4060), pan AKT (dilution 1:1000, Cat. # 4691) were obtained from Cell Signaling and actin (dilution 1:5000, Cat. # sc1616) was ordered from Santa Cruz Biotechnology, Inc. Membranes were washed five times with TBST, incubated with horseradish peroxide (HRP)-conjugated secondary antibodies in TBST/2% BSA for 1 h, rinsed with TBST, and detected by chemiluminescence (SuperSignal West Pico Chemiluminescent Substrate; Pierce). Actin-HRP antibody (Santacruz Biotech) was used to measure actin level as a loading control for each lane. To multiplex, the same membranes were used to probe multiple proteins with different molecular weights by carefully cutting the membranes horizontally at respective molecular weights as indicated by BioRad precision color marker which was run in the first lane. These cut membranes were separately probed with respective antibodies and finally assembled together and chemiluminescence was measured at different exposure times. The representative blots shown in the Supplementary Data 4 are indicated in the red box. For main figures, Fig. 2e and Fig. 3b, blots were cut from Supplementary Data 4.

### High complexity DNA barcode library

We used the Cell Tracker high complexity DNA barcode library BC13×13–30M-X (Cellecta, Inc, CA) containing more than 50 million unique barcodes. Each construct is comprised of a 267-bp-long oligonucleotide containing a 40-bp-long double barcode sequence (two 18-bp-long sequences independently and randomly selected from a defined dictionary of ~13,000 18nt barcode sequences and linked by a 4-nucleotide spacer) and a flanking primer pair for barcode amplification. The CellTracker 50 M Barcode Library was constructed in a Cellecta third-generation lentiviral pRSI16-U6-bc-HTS6-UbiC-TagRFP-2A-Puro-w vector that expresses both TagRFP (Evrogen) and a Puromycin resistance gene under a human Ubiquitin C promoter as selection markers for isolation of barcoded cell populations.

### Generation of barcoded MDA-MB-231 cell line

MDA-MB-231 cells were cultured in RPMI-1640 supplemented with 10%FBS and 1% penicillin-streptomycin. Cells were barcoded by mixing cell suspension, lentivirus, and 0.8 μg/ml polybrene. We infected 1.02 × 10^8^ cells with 14 µl of the virus with 6.7 × 10^8^ TU/ml viral titer targeting 10% of transduction for a multiplicity of infection (m.o.i.) of approximately 0.1 to ensure that the majority of cells were labeled with a single barcode per cell. We actually achieved 3% infectivity (3.06 × 10^6^ cells were barcoded out of 1.02 × 10^8^) based on the RFP-positive population analyzed by flow cytometry. Puromycin (1 µg/µl) selected barcoded cells were expanded to 9 × 10^7^ cells in culture for the minimal period to obtain a sufficient clonal representation and apportioned into 36 cryovials, each containing 2.5 × 10^6^ cells, for storage. For every experiment, a vial was thawed, and cells were expanded without discarding any cells until the population was enough for all replicates of all treatments.

### Treatment of DNA barcoded MDA-MB-231 cells

Two vials (V1 and V2) of DNA barcoded MDA-MB-231 cells were expanded to get enough cells and treated in parallel. Cells were treated with navitoclax to collect two replicates per vial (total 4 replicates) at each of 3 time-points, (i) baseline-S1, (ii) after 3 days of navitoclax 10 mM treatment and 10 days of recovery-S2, (iii) after 3 days of navitoclax 10 mM retreatment and 10 days of recovery-S3; Detailed description of DNA barcoded samples can be found in Supplementary Fig. 5a.

### Barcode amplification

Barcodes were amplified for next-generation sequencing (NGS) using two rounds of PCR with Titanium Taq DNA Polymerase (Clontech-Takara, Cat. no. 639208). Briefly, cells were pelleted by centrifugation and gDNA was extracted using Qiagen DNeasy blood and tissue kit (Cat. no. 69504) and stored at −80 °C. The first round of PCR reactions (18 cycles) was run using 5 µg of genomic DNA as a template. Parallel multiple PCR reactions (5 µg each) were run to include all of the DNA of the treatment or control group to avoid barcode loss. For the second round of PCR (14 cycles), P5 and P7 adapters that are complementary to immobilized primer sequences in the NGS Illumina flow cells and a 6-bp-long index sequence were added to the first-round PCR product. Each treatment or control group was labeled with one of the 16 unique indices to multiplex up to 16 samples for sequencing simultaneously. Products from the 2nd round of PCR were purified using QIAquick PCR purification kit (Cat no. 28104), resolved on a 2% agarose gel, and the 316-base pair band was excised and extracted using QIAquick Gel Extraction kit (Cat no. 28704).

### Next-generation barcode sequencing

NGS was carried out using the Illumina HiSeq platform at Yale Center for Genome Analysis. PCR-amplified products were analyzed using an Agilent Technologies 2100 bioanalyzer to determine the insert size. The concentrations were determined using qRT-PCR with a kit from Kapa Biosystems. PCR amplicons were prepared at 2 nM and loaded at 15 pM with 15% of spiked in PhiX Control (Illumina, Cat. no. FC-110-3001) and on the Illumina HiSeq2500 sequencer for single-end read in Rapid Mode using 50 TruSeq Rapid SBS Kit v2HS (cat no. FC-402-4022), TruSeq Rapid SR Cluster Kit v2-cBot-HS (Cat no. GD-402-4002) and HiSeq Rapid SR Flow Cell or High Output Mode using the 50 TruSeq SBS Kit v3HS (Cat no. FC-401-3002), TruSeq SR Cluster Kit v3-cBot-HS (Cat no. GD-401-3001) and HiSeq SR Flow Cell (Illumina). Samples were sequenced at 44 cycles for read 1 and 6 cycles for the I7 index read.

### Barcode data pre-processing

Barcode sequencing runs were converted to FASTQ files and analyzed using custom R scripts. First, reads were filtered by matching the 4-nucleotide spacer sequence (CGAA) that linked two 18-mer barcode sequences and the spacer sequence after the second barcode. The remaining reads were trimmed after the second spacer. Further, quality filtering was applied; reads with a Phred quality score <25 in more than 2 bp were removed, and then single bp with Phred quality score <25 were replaced with *N*. Next, two barcode sequences were extracted from each read and only reads with both barcodes from 16 to 20 bp long were matched against the barcodes dictionary provided by Cellecta. Matching to the dictionary was done sequentially: first allowing no mismatches and then increasing the number of allowed mismatches to two and matching the remaining reads. Reads with barcodes that did not match to dictionary were excluded. In all analyzed samples ~15% of reads were discarded during the pre-processing step.

### Merging replicated samples

Concordance between samples was assessed based on common barcodes that were visualized on Venn diagrams and quantified by Jaccard index, and by calculating the Pearson correlation coefficients between the abundance of common barcodes (Supplementary Fig. 5b). Next, all 4 replicates per treatment phase (2 biological replicates from experiment 1 and 2 from experiment 2) were merged, keeping all barcodes that were observed in at least 2 replicates (Supplementary Fig. 5b). Three merged samples (one sample per treatment phase) were used in further analysis.

### Calculation of barcode overlap between samples

All barcodes from 3 merged time points were divided into 7 groups based on co-occurrence patterns in the three treatment samples as visualized by Venn diagrams. We coded the groups of barcodes according to their existence in 3 subsequent time points, e.g., “100” is a group of barcodes that were found only in the baseline (first) time point. Barcodes prevalent in one sample (S5: experiment 2, measurement after 1st treatment), that probably arose during the PCR amplification step, and barcodes observed only after the 1st treatment (group 010) and not observed after 1st treatment (group 101) were considered technical artifacts and were not further analyzed. The reasons for excluding these two groups of barcodes are as follows. The proportion of the number of unique barcodes relative to the number of sequencing reads is much higher for sample S5 than other samples (Supplementary Fig. 6a). The concordance between biological replicates of S5 was much lower than for other samples, and many high abundance barcodes were observed only in one of the two biological replicates (Supplementary Fig. 6b). Furthermore, the proportion of barcodes observed only after the first treatment compared to all barcodes was considerably higher in biological replicate 2 (group 010; Supplementary Fig. 6c). There were 3416 low abundance barcodes observed in the baseline sample and after the second treatment (group 101; Supplementary Fig. 6d). Since these last two groups of barcodes were not biologically interesting and are suspected to be likely technical artifacts, we removed them from further analysis. Abundance-weighted barcode prevalence was visualized using clonal evolution plots from Timescape R package^47^.

### Single-cell RNA sequencing on 10X Chromium platform

MDA-MB-231 cells were treated using two cycles of navitoclax. Samples at the following four times were collected for single-cell analysis: (i) baseline-S1, (ii) after 3 days of 10 μM navitoclax treatment-S2, (iii) after 10 days of drug holiday-S3, (iv) after 3 days of 10 μM navitoclax retreatment-S4. The entire experiment lasted 18 days. Immediately after plate harvesting, cells were trypsinized and a single cell suspension of 6000 cells per sample at 1000 cells/ml with viability above 90% were processed at YCGA on the 10X Chromium following the standard manufacturer protocol. For sequencing, two samples were multiplexed on one lane of a HiSeq 4000 flow cell giving 25,000 reads per cell. Chromium Single Cell 3′ Library and Gel Bead Kit V2 (PN-120237), Chromium Single Cell A Chip Kit (PN-120236), and Chromium i7 Multiplex Kit (PN-120262) were used to prepare the single-cell library following the manufacturer’s instructions. The Cell Ranger Single-Cell Software suite^48^ from 10X Genomics was used for demultiplexing, cell barcode processing, and gene-level quantification of the raw scRNAseq profiles.

### Single-cell RNA data pre-processing

For each sample, low-quality cells were filtered by thresholding on the number of expressed genes (bottom 5% removed), total UMI counts (top 1% removed), and percent of expression of mitochondrial genes (cells with more than 15% of mitochondrial genes removed). In addition, a model of correspondence between the number of expressed genes and total UMI counts was fitted using smoothing splines. Cells outlying from the model (|*z*-score|> 3) were excluded. In this step, for each sample, ~7% of the cells were filtered. Next, genes expressed in less than 1% of cells were filtered, leaving ~12,000 genes in the analysis. Data were normalized using the modified median-of-ratios method from DESeq2 R package^49^. The main idea of the modification was to use only expressed cells in the calculation of reference sample and calculation of median of expressed genes for each cell, due to the sparsity of single-cell data. At last, normalized data were log2-transformed (log2(*x* + 1) transformation).

### Modeling of single-cell expression data

A stochastic dropout effect (no expression), caused by either technical or biological factors, is widely observed in single-cell experiments. Thus, we used a two-part generalized linear model that simultaneously models the rate of expression over the background of various transcripts and the positive expression mean as implemented in the MAST R package^50^. We adjusted the model for two covariates: cellular detection rate (the fraction of genes that are detectably expressed in each cell) and percent of expression of mitochondrial genes per cell. Applying the model to single-cell expression data leads to an estimation of log odds ratio (logOR), quantifying the ratio (fold) of dropout odds (probability) between the two conditions, and log fold changes (logFC), quantifying the mean difference in gene expression between cells in the two conditions. Statistical significance of these measures was calculated using the Wald test with Bonferroni correction for multiple testing to get adjusted *p*-values. We used logFC and corresponding adjusted *p*-values to find differentially expressed genes between subsequent treatment phases and logOR to find the markers of cell groups defined in the DNA barcoding experiment. We summarized logFC estimates on gene-set level using a collection of MSigDB Hallmark pathways^51^. Residuals from the MAST model, that provide the scaled and centered expression data for each gene after removing the contribution of the unwanted covariates, were further used for clustering of the cells.

### Sample alignment and clustering

First, we selected the highly variable genes per sample by analyzing residuals from the smooth-spline model of the association between gene expression mean and variance on a log2 scale. The filtering was applied, and the model was fitted repeatedly until no outliers (|*z*-score| >3) were found. Next, 4 samples from the same experiment were integrated based on a common source of gene variation using Seurat package^52^. Briefly, to reduce data dimensionality and find shared correlation patterns between samples canonical correlation analysis (CCA) was performed. The First 10 CCA components, as recommended by ‘MetageneBicorPlot’ function from the Seurat package, were used to align samples. Prior to alignment, 10% of cells with the lowest ratio of variance explained by PCA to CCA per sample, were removed as they may represent non-overlapping cells. The CCA basis vectors were aligned between samples by Seurat, resulting in a single, integrated dataset. Then, clusters of cells were identified by a shared nearest neighbor modularity optimization-based clustering algorithm. The resolution parameter that sets the ‘granularity’ of the downstream clustering was optimized by finding knee-point on the within-sum-of-squares vs. the number of clusters plot, giving 7 final clusters. Each cluster was labeled based on its cell composition, e.g., for cluster S1 more than half of the cells are from sample S1. After alignment and clustering, cells were visualized using UMAP with cosine distance metric^53^, the number of nearest neighbors set to 10, and the minimum distance between points set to 0.

### Reporting summary

Further information on research design is available in the Nature Research Reporting Summary linked to this article.

## Supplementary information

Supplementary Information

Supplementary Data 1

Supplementary Data 2

Supplementary Data 3

Reporting Summary

## Data Availability

The data generated and analyzed during this study are described in the following data record: 10.6084/m9.figshare.14362850^54^. All DNA barcoding data and single-cell sequencing data generated in this study have been deposited in the Sequence Read Archive under project accession number https://identifiers.org/ncbi/insdc.sra:SRP259903 (BioProject accession: PRJNA630413)^55^. The data underlying Supplementary Fig. 3 Panels A & B are openly available from the Cancer Cell Line Encyclopedia (CCLE) at https://portals.broadinstitute.org/ccle. Other data files underlying the related manuscript are shared openly as part of the data record^54^.

## Code availability

*R* scripts implementing the custom pipeline to load and process DNA barcoding sequencing data for extracting and summarizing the unique barcodes are available at https://github.com/mmarczyk363/DNA_Barcodes.
