# Supplementary material for: Treatment scheduling effects on the evolution of drug resistance in heterogeneous cancer cell populations
Source: NPJ Breast Cancer. 2021 May 26;7:60. doi: 10.1038/s41523-021-00270-4 (PMC8154902; doi:10.1038/s41523-021-00270-4)
Supplement: Supplementary file 1 — Supplementary Information [file 41523_2021_270_MOESM1_ESM.pdf]

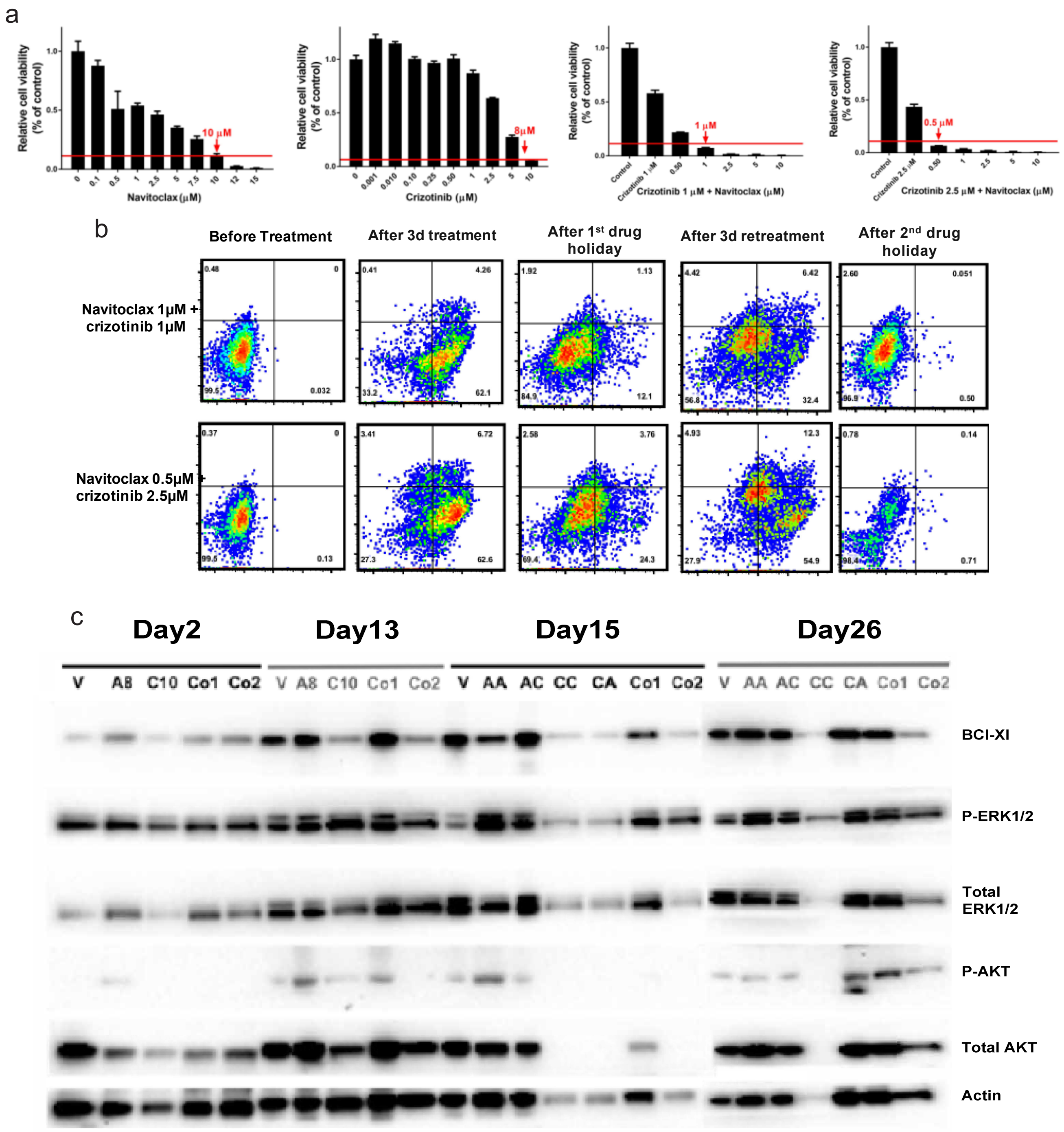

Supplementary Figure 1. Navitoclax and Crizotinib Alone and in Combination in MDA-MB-231. (a) IC_{90} doses of crizotinib and navitoclax alone and in combination. For IC_{90} calculation, at least three independent experiments were done, each in triplicate and IC_{90} was calculated as a dose where 90% of cells were killed compared to vehicle at the end of 72h. (b) Dot plots for joint annexin V-FITC (x-axis) and PI staining (y-axis) showing percentage of apoptotic cells. Flow cytometry was done on concurrent combination treatment regimens of combination 1 navitoclax 1μM + crizotinib 1μM and combination 2 navitoclax 0.5μM + crizotinib 2.5μM. The schedules included two cycles, each consisting of a 3-day treatment period followed by a 10-day recovery period. Samples were collected at time points: pre-treatment baseline; after 3 days treatment; after 10 days of recovery; after 3 days of treatment in cycle 2; after 10 days of recovery in cycle 2. (c) Western blot for sequential and concurrent treatment regimens of navitoclax and crizotinib at indicated time points of longest treatment schedule.
.

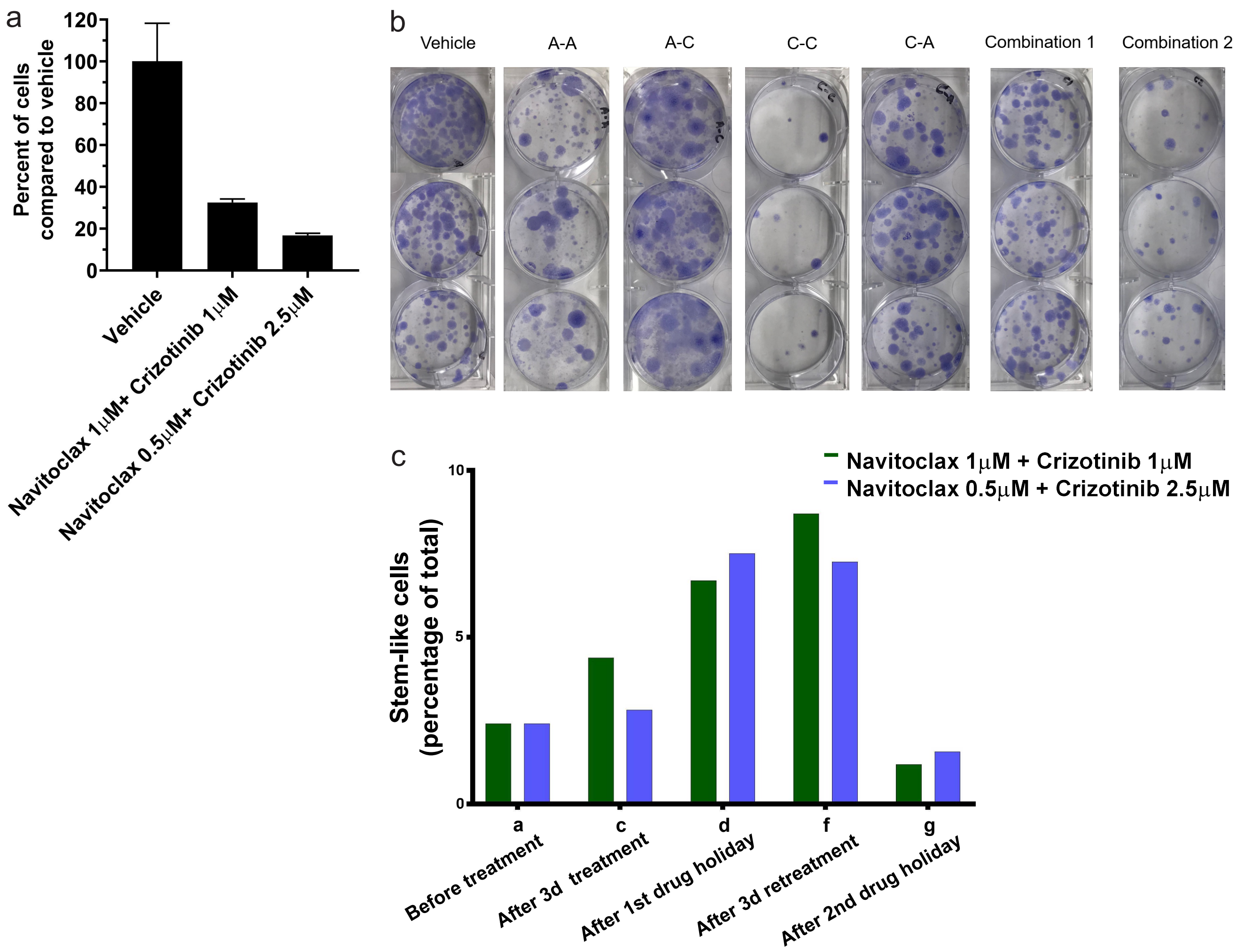

Supplementary Figure 2. Cell Growth of Navitoclax and Crizotinib Concurrent Treatment Compared to Vehicle and Colony Formation Assay. (a) Cell growth of navitoclax and crizotinib combination 1 and combination 2 compared to vehicle after 72h of treatment. At least three independent experiments were done, each in triplicate. (b) Representative pictures of colony formation assay for sequential and concurrent treatments. Assay was done in triplicate by plating cells after the end of 26-day schedule for each treatment. (c) Percentage of stem-like cells measured by flow cytometry using cell surface markers EpCAM, CD24 and CD44 at different treatment points.

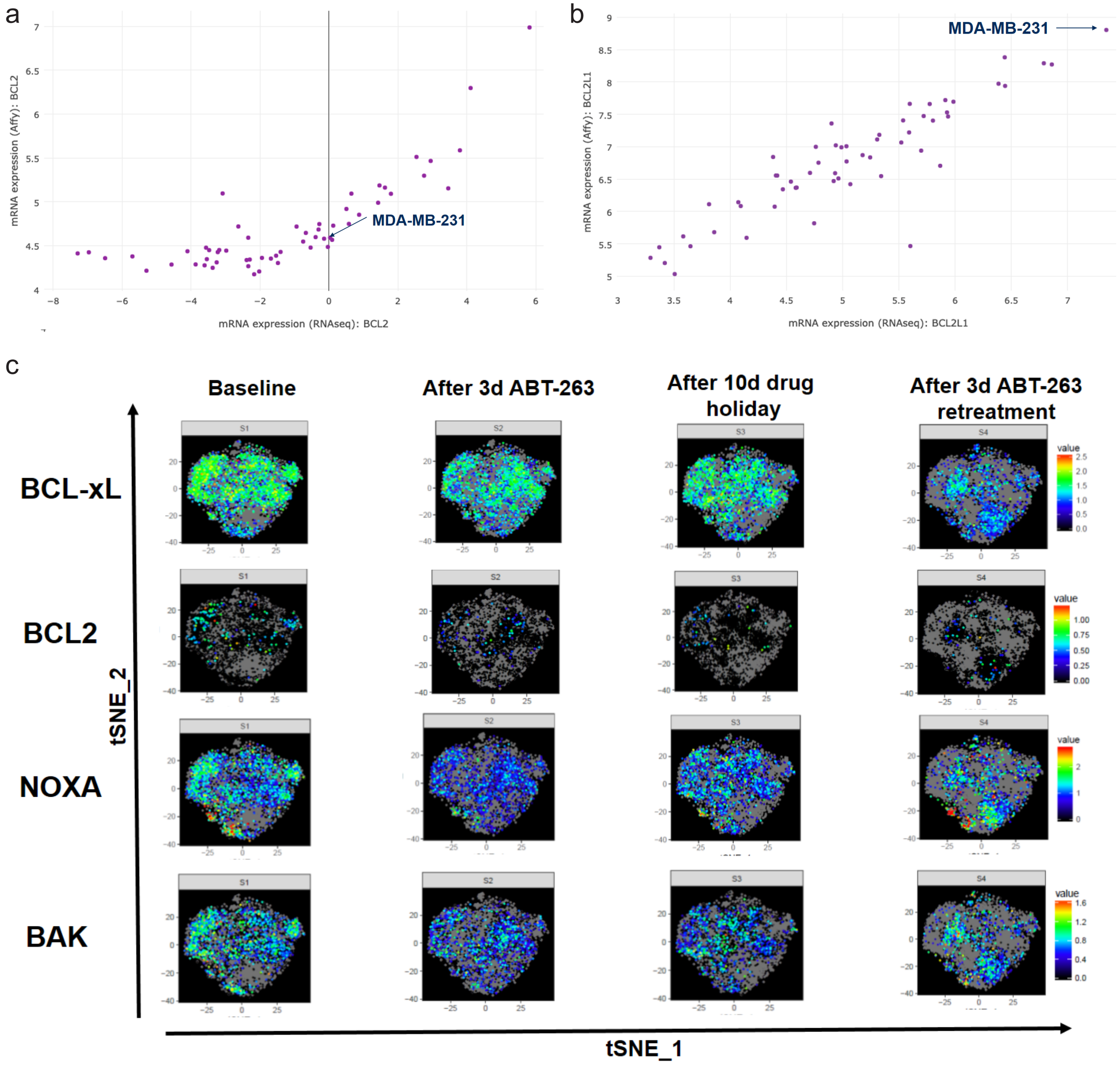

Supplementary Figure 3. Expression of Bcl Family mRNA in MDA-MB-231. (a) Expression of BCL2 in cell lines in Cancer Cell Line Encyclopedia (CCLE). (b) Expression of BCL2L1 encoding for Bcl-xl in CCLE cell lines. (c) Single cell RNAseq analysis of MDA-MB-231 cells at baseline and after two subsequent cycles of navitoclax. BCL2L1 (labeled as BCL-xL) is highly expressed at baseline and appears slightly reduced at the end of the first treatment cycle. BCL2 basal expression is very low and is expressed in small minority of the cells. Treatment appears to further reduce BCL2 levels.

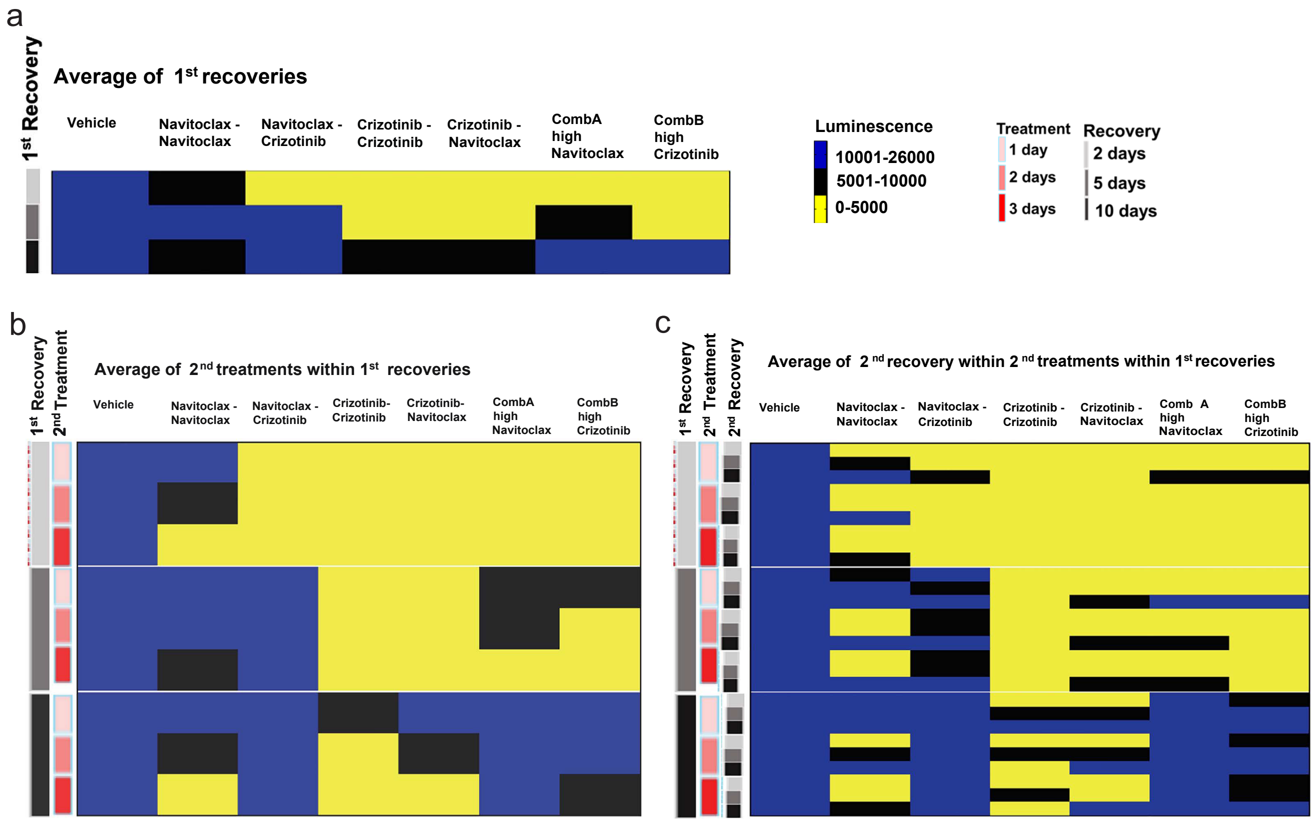

Supplementary Figure 4. Effect of Treatment Scheduling on Cell Growth. (a) Effect of 1st drug holiday. (b) Effect of retreatment within 1st recovery. (c) Effect of 2nd drug holiday in retreatment and 1st drug holiday.

a

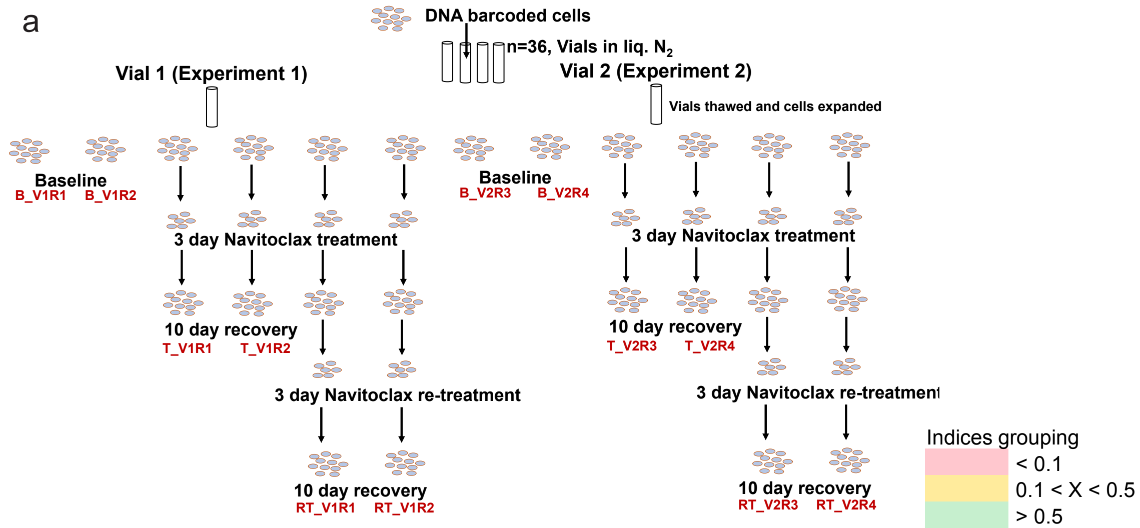

b

Jaccard index (upper triangle) and Pearson correlation (lower triangle) for concordance between replicates

Sample	B_V1R1	B_V1R2	T_V1R1	T_V1R2	RT_V1R1	RT_V1R2	B_V2R3	B_V2R4	T_V2R3	T_V2R4	RT_V2R3	RT_V2R4
B_V1R1	-	**0.568**	0.719	0.606	0.432	0.392	0.120	0.129	0.123	0.127	0.102	0.088
B_V1R2	**-0.347**	-	0.660	0.554	0.395	0.357	0.124	0.130	0.129	0.133	0.103	0.087
T_V1R1	-0.146	-0.172	-	**0.766**	0.563	0.513	0.132	0.142	0.136	0.140	0.111	0.094
T_V1R2	-0.136	-0.225	**0.950**	-	0.570	0.540	0.123	0.135	0.127	0.129	0.109	0.094
RT_V1R1	-0.019	-0.035	0.197	0.175	-	**0.544**	0.110	0.125	0.113	0.114	0.106	0.094
RT_V1R2	-0.118	-0.171	0.890	0.849	**0.291**	-	0.106	0.122	0.108	0.108	0.102	0.090
B_V2R3	0.051	0.109	0.043	0.051	0.002	0.064	-	**0.455**	0.357	0.509	0.349	0.293
B_V2R4	0.058	0.121	0.023	0.027	0.003	0.062	**-0.301**	-	0.347	0.421	0.340	0.319
T_V2R3	0.073	0.108	0.409	0.423	0.092	0.404	-0.032	0.018	-	**0.454**	0.275	0.255
T_V2R4	0.068	0.093	0.424	.434	0.093	0.406	-0.082	0.027	**0.625**	-	0.374	0.274
RT_V2R3	0.014	0.009	0.078	0.070	0.213	0.308	-0.007	-0.007	0.172	0.167	-	**0.336**
RT_V2R4	0.016	0.008	0.081	0.072	0.210	0.315	-0.021	-0.011	0.173	0.185	1.000	-

c

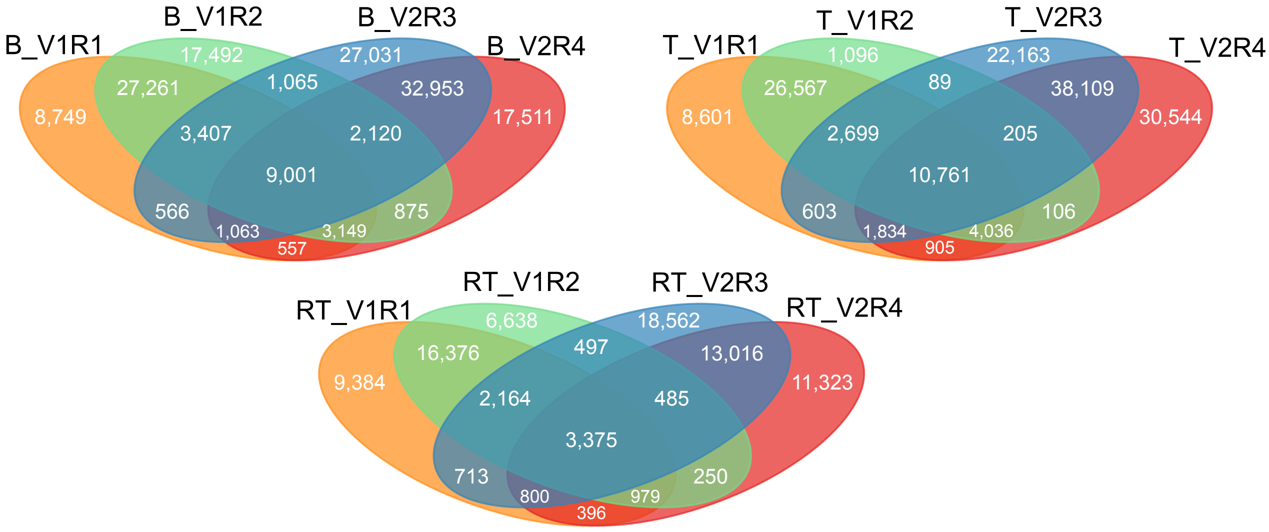

Supplementary Figure 5. Merging of Replicates from Vials in DNA-barcoding Experiment. (a) Detailed description of samples used in the experiment. (b) Jaccard index and Pearson correlation coefficient for comparison of concordance between samples. (c) The number of unique barcodes common between samples within the same treatment phase.

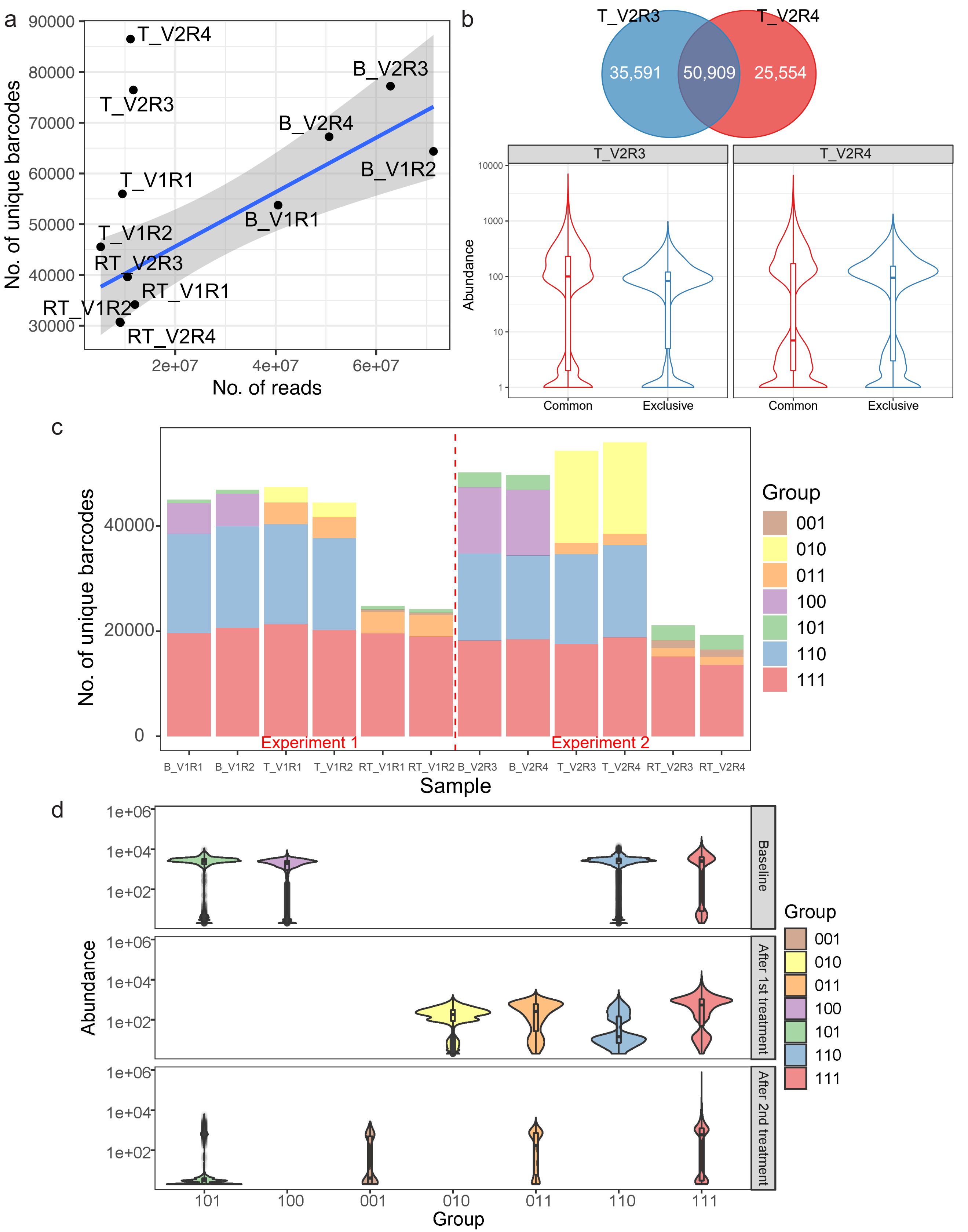

Supplementary Figure 6. Explanation for Excluding Two Groups of Barcodes from Main Analysis. (a) Association between no. of sequencing reads and observed no. of unique barcodes per sample. (b) Barcode concordance and abundance between two replicates of after treatment sample from vial 2. (c) Distribution of barcodes divided into 7 groups of cells in two vials. (d) Abundance of barcodes divided into 7 groups after experiments merging.

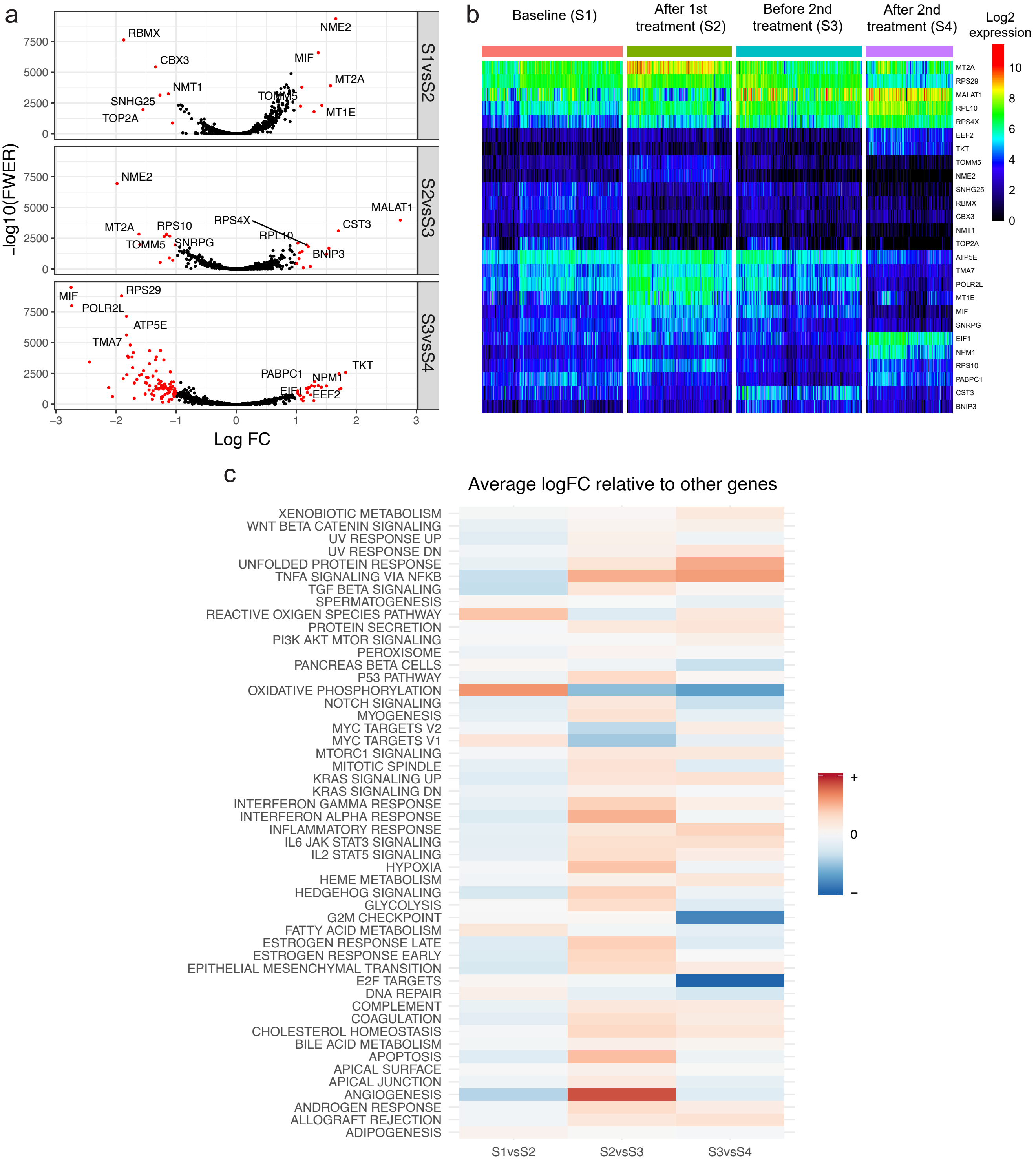

Supplementary Figure 7. Gene Expression Change in Subsequent Treatment Steps. (a) Volcano plots presenting significant genes with |FC| > 1 (red dots). Among them, 5 with the lowest p-value on both ends are annotated. (b) Expression heatmap for genes annotated on panel A. (c) Expression change between subsequent treatment steps summarized on pathway level.

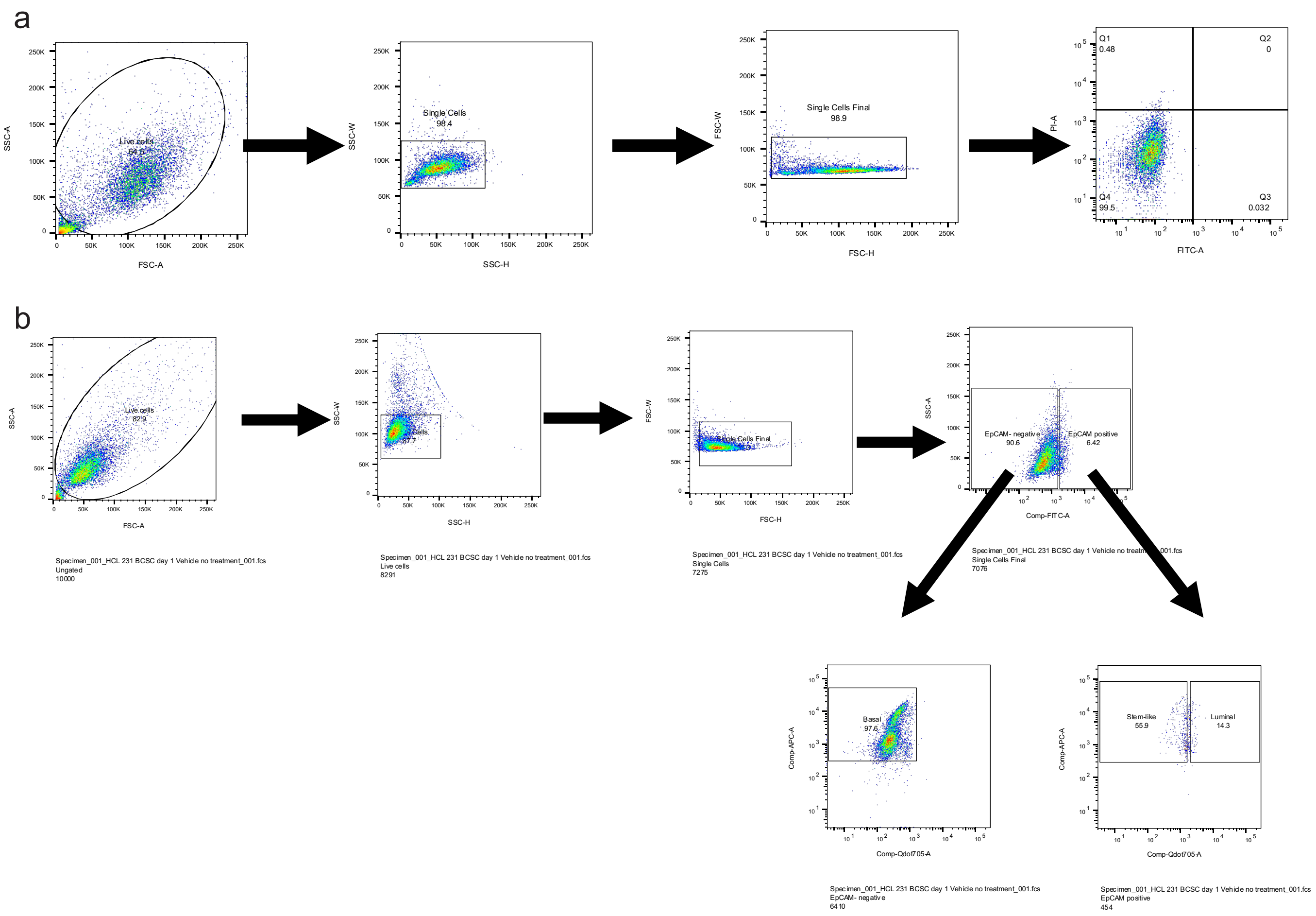

Supplementary Figure 8. Gating Strategy for flowcytometry analysis. (a) Apoptotic cells (b) Breast cancer stem-like cells.

Supplementary Data 4

Uncropped blots

Western Blot Antibodies Details

Sr. No.	Target	Product detail	Mol. Wt.	Source	Supplier	Cat. No.	Lot. No.
1	PARP		89, 116	R mAb	Cell Signaling	9542	13
2	Bcl-xL	54H6	30	R mAb	Cell Signaling	2764	6
3	Phospho-P44/42 MAPK (Erk1/2)	Thr202/Tyr204-D13.14.4E	42,44	R mAb	Cell Signaling	4370	17
4	P44/42 MAPK (Erk1/2)	137F5	42,44	R mAb	Cell Signaling	4695	21
5	pAKT	Ser473-D9E	60	R mAb	Cell Signaling	4060	16
6	Akt (pan)	C67E7	60	R mAb	Cell Signaling	4691	20
7	Actin	I-19	43	Goat Polyclonal	Santacruz Biotechnology	sc1616	H2907

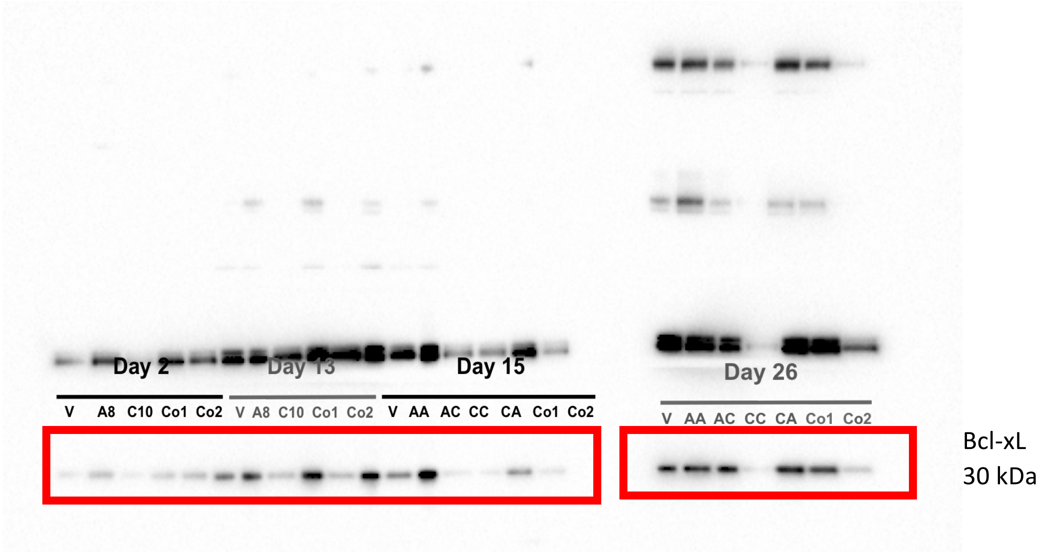

The representative blots shown in the Supplementary Fig. 1c are indicated in red box.

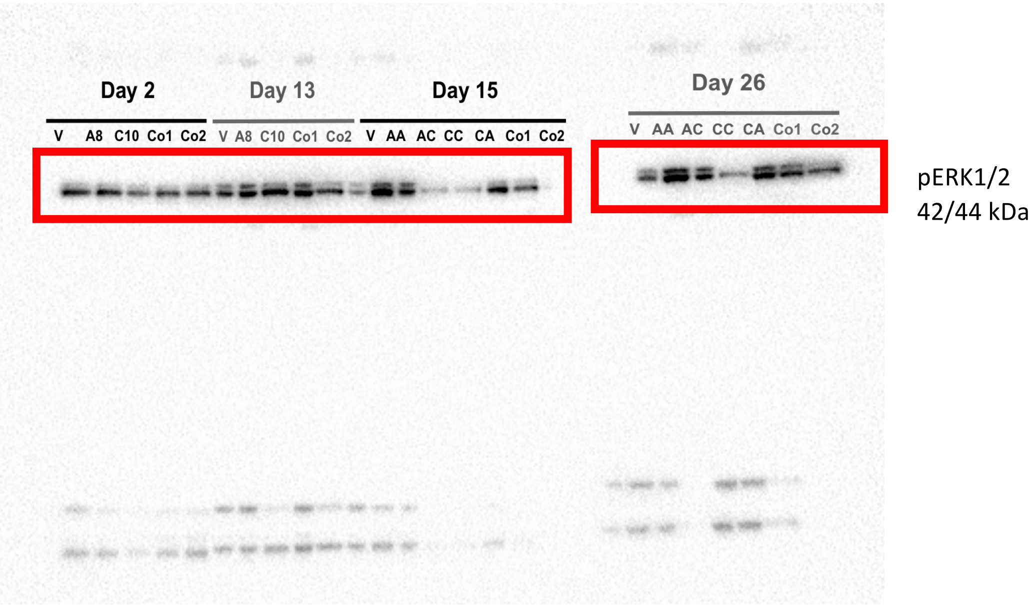

The representative blots shown in the Supplementary Fig. 1c are indicated in red box.

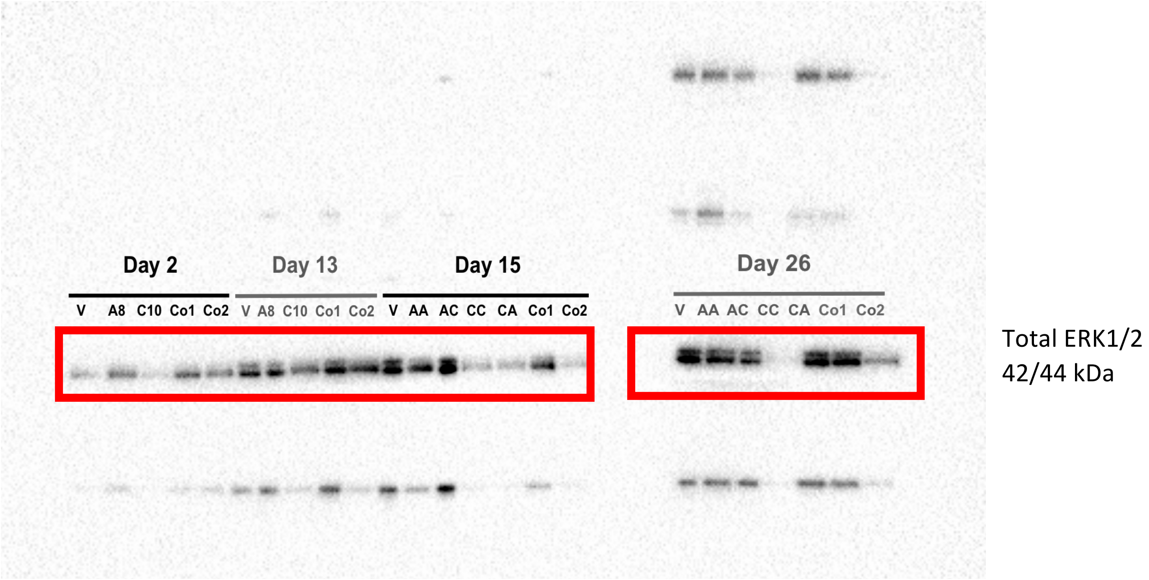

The representative blots shown in the Supplementary Fig. 1c are indicated in red box.

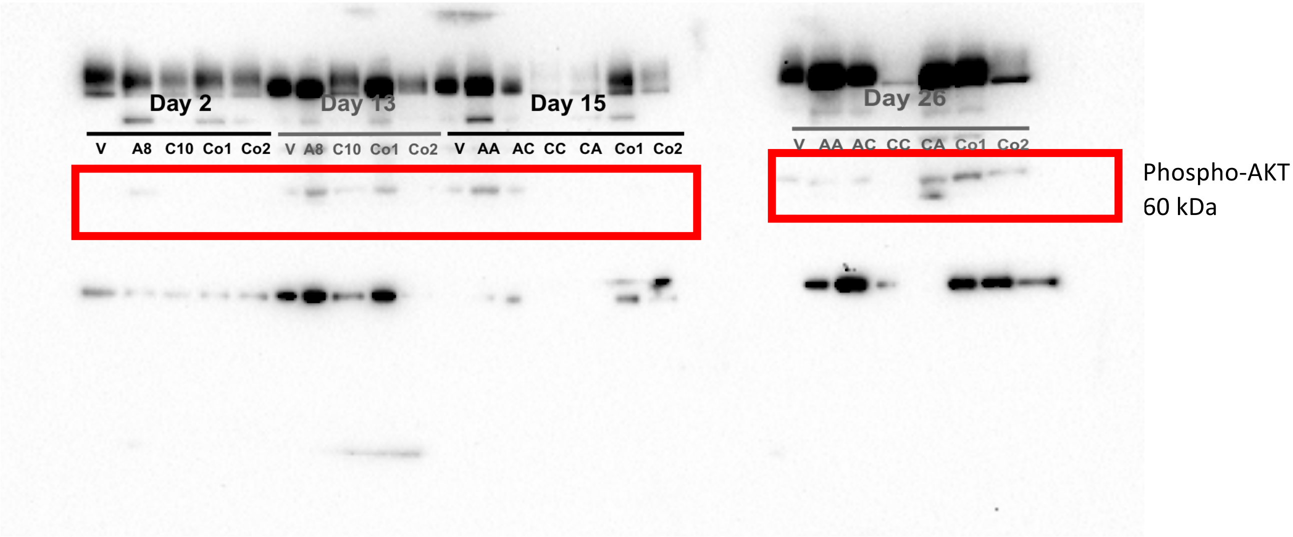

The representative blots shown in the Supplementary Fig. 1c are indicated in red box.

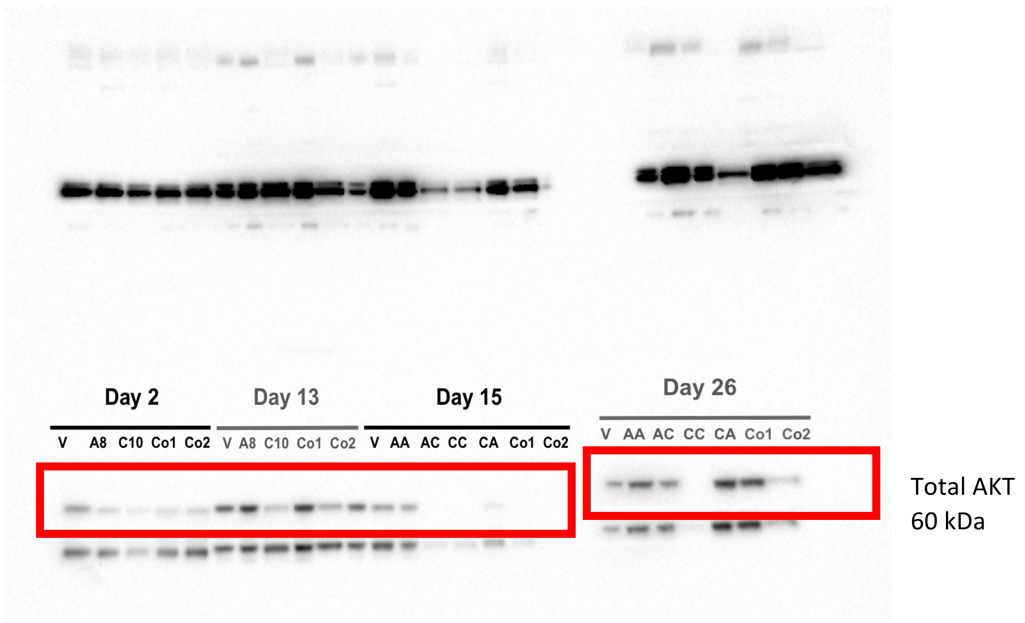

The representative blots shown in the Supplementary Fig. 1c are indicated in red box.

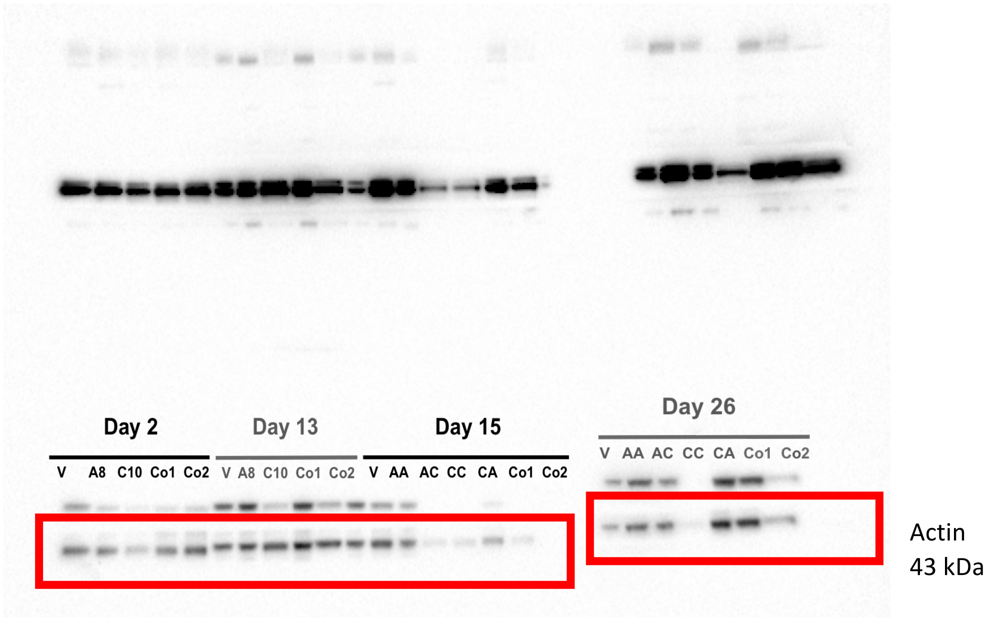

The representative blots shown in the Supplementary Fig. 1c are indicated in red box.

Other supplementary data

Supplementary data 1 - Cell-titer glow readings for all treatment schedules for each treatment regimen.

Supplementary data 2 - Calibration curve: number of cells plated on day 0 versus cell-titer glow readings on 0, 24, 48, 72h of treatment for each regimen.

Supplementary data 3 - Densitometry analysis for Western blot bands.
