# Supplementary material for: Treatment scheduling effects on the evolution of drug resistance in heterogeneous cancer cell populations
Source: NPJ Breast Cancer. 2021 May 26;7:60. doi: 10.1038/s41523-021-00270-4 (PMC8154902; doi:10.1038/s41523-021-00270-4)
Supplement: Supplementary file 5 — Reporting Summary [file 41523_2021_270_MOESM5_ESM.pdf]

nature research

Corresponding author(s): Christos Hatzis

Last updated by author(s): Oct 21, 2020

Reporting Summary

Nature Research wishes to improve the reproducibility of the work that we publish. This form provides structure for consistency and transparency in reporting. For further information on Nature Research policies, see our Editorial Policies and the Editorial Policy Checklist.

Statistics

For all statistical analyses, confirm that the following items are present in the figure legend, table legend, main text, or Methods section.

n/a	Confirmed	
☐	☒	The exact sample size (n) for each experimental group/condition, given as a discrete number and unit of measurement
☐	☒	A statement on whether measurements were taken from distinct samples or whether the same sample was measured repeatedly
☐	☒	The statistical test(s) used AND whether they are one- or two-sided<br>*Only common tests should be described solely by name; describe more complex techniques in the Methods section.*
☐	☒	A description of all covariates tested
☐	☒	A description of any assumptions or corrections, such as tests of normality and adjustment for multiple comparisons
☒	☐	A full description of the statistical parameters including central tendency (e.g. means) or other basic estimates (e.g. regression coefficient) AND variation (e.g. standard deviation) or associated estimates of uncertainty (e.g. confidence intervals)
☐	☒	For null hypothesis testing, the test statistic (e.g. F, t, r) with confidence intervals, effect sizes, degrees of freedom and P value noted<br>*Give P values as exact values whenever suitable.*
☒	☐	For Bayesian analysis, information on the choice of priors and Markov chain Monte Carlo settings
☒	☐	For hierarchical and complex designs, identification of the appropriate level for tests and full reporting of outcomes
☒	☐	Estimates of effect sizes (e.g. Cohen's d, Pearson's r), indicating how they were calculated

Our web collection on statistics for biologists contains articles on many of the points above.

Software and code

Policy information about availability of computer code

Data collection	No specific software for data collection was used
Data analysis	DNA barcode data analysis:<br><br>R programming language v.3.6.1<br>Custom R scripts to load and pre-process DNA barcoding data. Available here: https://github.com/mmarczyk363/DNA_Barcodes<br>Timescape R package v. 1.8.0<br><br>Single-cell 10x data analysis:<br><br>Cell Ranger Single-Cell Software suite v. 2.1.0<br>FastQC software v. 0.11.5<br>R programming language v.3.6.1<br>DESeq2 R package v. 1.24.0<br>MAST R package v. 1.10.0<br>Seurat R package v. 2.3<br>UMAP R package v. 0.2.5.0

For manuscripts utilizing custom algorithms or software that are central to the research but not yet described in published literature, software must be made available to editors and reviewers. We strongly encourage code deposition in a community repository (e.g. GitHub). See the Nature Research guidelines for submitting code & software for further information.

Data

Policy information about availability of data

All manuscripts must include a data availability statement. This statement should provide the following information, where applicable:

- Accession codes, unique identifiers, or web links for publicly available datasets
- A list of figures that have associated raw data
- A description of any restrictions on data availability

Provide your data availability statement here.

Field-specific reporting

Please select the one below that is the best fit for your research. If you are not sure, read the appropriate sections before making your selection.

☒ Life sciences ☐ Behavioural & social sciences ☐ Ecological, evolutionary & environmental sciences

For a reference copy of the document with all sections, see nature.com/documents/nr-reporting-summary-flat.pdf

Life sciences study design

All studies must disclose on these points even when the disclosure is negative.

Sample size	*Describe how sample size was determined, detailing any statistical methods used to predetermine sample size OR if no sample-size calculation was performed, describe how sample sizes were chosen and provide a rationale for why these sample sizes are sufficient.*
Data exclusions	*Describe any data exclusions. If no data were excluded from the analyses, state so OR if data were excluded, describe the exclusions and the rationale behind them, indicating whether exclusion criteria were pre-established.*
Replication	*Describe the measures taken to verify the reproducibility of the experimental findings. If all attempts at replication were successful, confirm this OR if there are any findings that were not replicated or cannot be reproduced, note this and describe why.*
Randomization	*Describe how samples/organisms/participants were allocated into experimental groups. If allocation was not random, describe how covariates were controlled OR if this is not relevant to your study, explain why.*
Blinding	*Describe whether the investigators were blinded to group allocation during data collection and/or analysis. If blinding was not possible, describe why OR explain why blinding was not relevant to your study.*

Reporting for specific materials, systems and methods

We require information from authors about some types of materials, experimental systems and methods used in many studies. Here, indicate whether each material, system or method listed is relevant to your study. If you are not sure if a list item applies to your research, read the appropriate section before selecting a response.

Materials & experimental systems

n/a	Involved in the study	
☐	☒	Antibodies
☐	☒	Eukaryotic cell lines
☒	☐	Palaeontology and archaeology
☒	☐	Animals and other organisms
☒	☐	Human research participants
☒	☐	Clinical data
☒	☐	Dual use research of concern

Methods

n/a	Involved in the study	
☒	☐	ChIP-seq
☐	☒	Flow cytometry
☒	☐	MRI-based neuroimaging

Antibodies

Antibodies used

Western blot antibodies:
PARP (Cell Signaling, Cat# 9542, Lot 13)
Bcl-xL (54H6, Cell Signaling, Cat#2764, Lot 6)
Phospho-P44/42 MAPK (Erk1/2)(Thr202/Tyr204-D13.14.4E, Cell Signaling, Cat# 4370, Lot 17)
Phospho-P44/42 MAPK (Erk1/2)(137F5, Cell Signaling, Cat# 4695, Lot 21)
pAKT (Ser473-D9E, Cell Signaling, Cat#4060, Lot 16)
Akt pan (C67E7, Cell Signaling, Cat# 4691, Lot 20)
Actin (i-19, Santacruz Biotechnology, Cat# sc1616, Lot H2907)

Validation | Data provided in Supplementary Information

Eukaryotic cell lines

Policy information about cell lines

Cell line source(s)	MDA-MB-231 cell line was purchased from ATCC (ATCC HTB-26)
Authentication	Cell line was authenticated by ATCC by short-tandem repeat profiling, karyotyping, morphology, and cytochrome C oxidase I testing. Cell line was used at passages 3 to 9, and cultured less than 3 months after thawing
Mycoplasma contamination	Cell line was not tested for mycoplasma contamination. Cell lines were used between 3-9 passages.
Commonly misidentified lines (See ICLAC register)	*Name any commonly misidentified cell lines used in the study and provide a rationale for their use.*

Flow Cytometry

Plots

Confirm that:

☒ The axis labels state the marker and fluorochrome used (e.g. CD4-FITC).

☒ The axis scales are clearly visible. Include numbers along axes only for bottom left plot of group (a 'group' is an analysis of identical markers).

☒ All plots are contour plots with outliers or pseudocolor plots.

☒ A numerical value for number of cells or percentage (with statistics) is provided.

Methodology

Sample preparation	For apoptotic cell assessment, floating cells were combined with adherent cells harvested by trypsinization and washed with PBS. Annexin V binding buffer, Annexin V, and Propidium Iodide (PI) were added to cells and incubated for 15 min in dark at room temperature. Samples were analyzed with the BD LSRII flow cytometer, by recording 10,000 events per sample.<br>For stem-like cell assessment, cells were dissociated using 0.25% Trypsin/EDTA and washed with PBS. Cells were then spun down and approximately 1 * 106 cells were resuspended in 100ul volume of blocking buffer (1xDPBS + 2% BSA) for 5 min. Then 20 µl of each of primary antibodies, APC Mouse Anti-Human CD44 (Cat. # 560890 BD Pharmingen), PE Mouse Anti-Human CD24 (Cat. # 560991 BD Pharmingen) and ESA/Ep-CAM, Mouse MAb anti-Human (Cat. # BMDMM101416 accurate chemical and scientific corporation) were added to cells and gently mixed. Cells were incubated with Abs at room temperature for 20 min mixing at every 5 min interval and then quenched with 1ml Blocking Buffer and spun down at 1200 rpm for 5 min. Supernatant was aspirated , and cells were resuspended in 1000µl of PBS keep on ice until analyzed by flow cytometer.
Instrument	BD Biosciences LSRII
Software	FlowJo version 7.6.5
Cell population abundance	Cells were not sorted and processed as per protocol above
Gating strategy	For apoptotic cell assessment, the starting live population was identified by gating FSC/SSC and doublets were excluded by SSC-H/SSC-W and FSC-H/FSC-W. Cells were then identified as apoptotic cells that are Annexin V positive/PI negative and Annexin V -PI double positive. Gates were applied using unstained cells as controls.<br>For cancer stem-like cell assessment, the starting live population was identified by gating FSC/SSC and doublets were excluded by SSC-H/SSC-W and FSC-H/FSC-W. Cells were then identified as EpCAM-FITC negative (below 103) EpCAM-FITC negative (beyond 103). Basal cells were identified as EpCAM- CD44+CD24- from EpCAM negative cell population. Stem-like cells were identified as EpCAM+CD44+CD24- and luminal cells were identified as EpCAM+CD44+CD24+ from EpCAM positive cell population. Gates were applied using IgG and unstained cells as controls.

☒ Tick this box to confirm that a figure exemplifying the gating strategy is provided in the Supplementary Information.
